# Supplementary material for: A model for drug transport across two membranes of Gram-negative bacteria by an MFS tripartite assembly
Source: Nat Commun. 2026 Mar 16;17:4039. doi: 10.1038/s41467-026-70500-5 (PMC13139468; doi:10.1038/s41467-026-70500-5)
Supplement: Supplementary file 1 — Supplementary Information [file 41467_2026_70500_MOESM1_ESM.pdf]

SUPPLEMENTARY INFORMATION

A model for drug transport across two membranes of Gram-negative bacteria by an MFS tripartite assembly

Zhaojun Zhong[1*], Tuerxunjiang Maimaiti[1*], Matthew L. Jackson[2*], Rui Dong[3*], Xueyan Gao[1*], Qing Ouyang[1], Wenqian Wang[1], Jinliang Guo[1], Shangrong Li[1], Wenyu Shang[1], Huajun Liu[1], Hongnian Jiang[3], Shuo Zhang[3], Ulrich Zachariae [4**], Ben F. Luisi [2**], Yanjie Chao [3**], Dijun Du[1**]

[1]School of Life Science and Technology, ShanghaiTech University, Shanghai, China
[2] Department of Biochemistry, University of Cambridge, Cambridge, UK
[3] Shanghai Institute of Immunity and Infection, Chinese Academy of Sciences, Shanghai, China
[4] Molecular Biophysics, Biological Chemistry and Drug Discovery, School of Life Sciences, University of Dundee, UK
[*] These authors contributed equally.
[**]Corresponding Authors: dudj@shanghaitech.edu.cn; yjchao@ips.ac.cn; bfl20@cam.ac.uk; u.zachariae@dundee.ac.uk

Supplementary Figures 1–23 and Supplementary Tables 1 and 2.

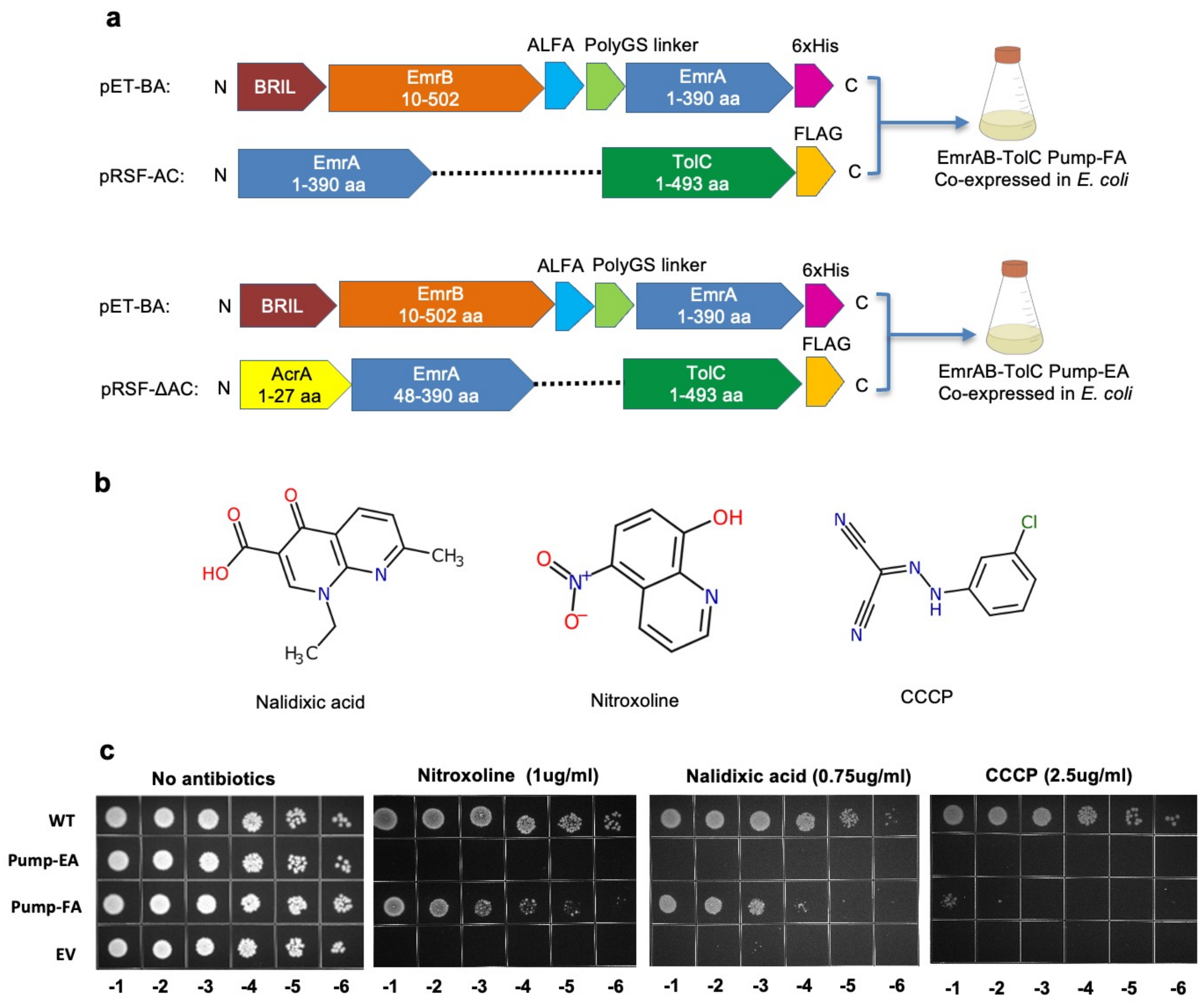

Supplementary Figure 1 | Drug efflux activity of EmrAB-TolC tripartite pumps confers antibiotic resistance. **a,** Construction of vectors for overexpression of EmrAB-TolC complexes. **b,** The chemical structures of Nalidixic acid, Nitroxoline and CCCP (Carbonyl Cyanide m-Chlorophenyl Hydrazone). **c,** Antibiotic sensitivity assays of cells expressing the wild-type EmrAB-TolC pump, pump-EA and pump-FA. The *E. coli* C43(DE3) Δ*acrAB* cells expressing wild-type EmrAB-TolC pump, engineered pump-EA and pump-FA, respectively, from plasmids were grown in LB medium to OD_{600} of 0.5, then 10-fold serially diluted (Column -1 to -6) and plated on LB agar containing 60 μM IPTG in the presence of drugs. Cell growth was monitored after culture at 37°C overnight. "WT" and "EV" denote wild-type EmrAB-TolC pump and the empty vector containing the negative control, respectively.

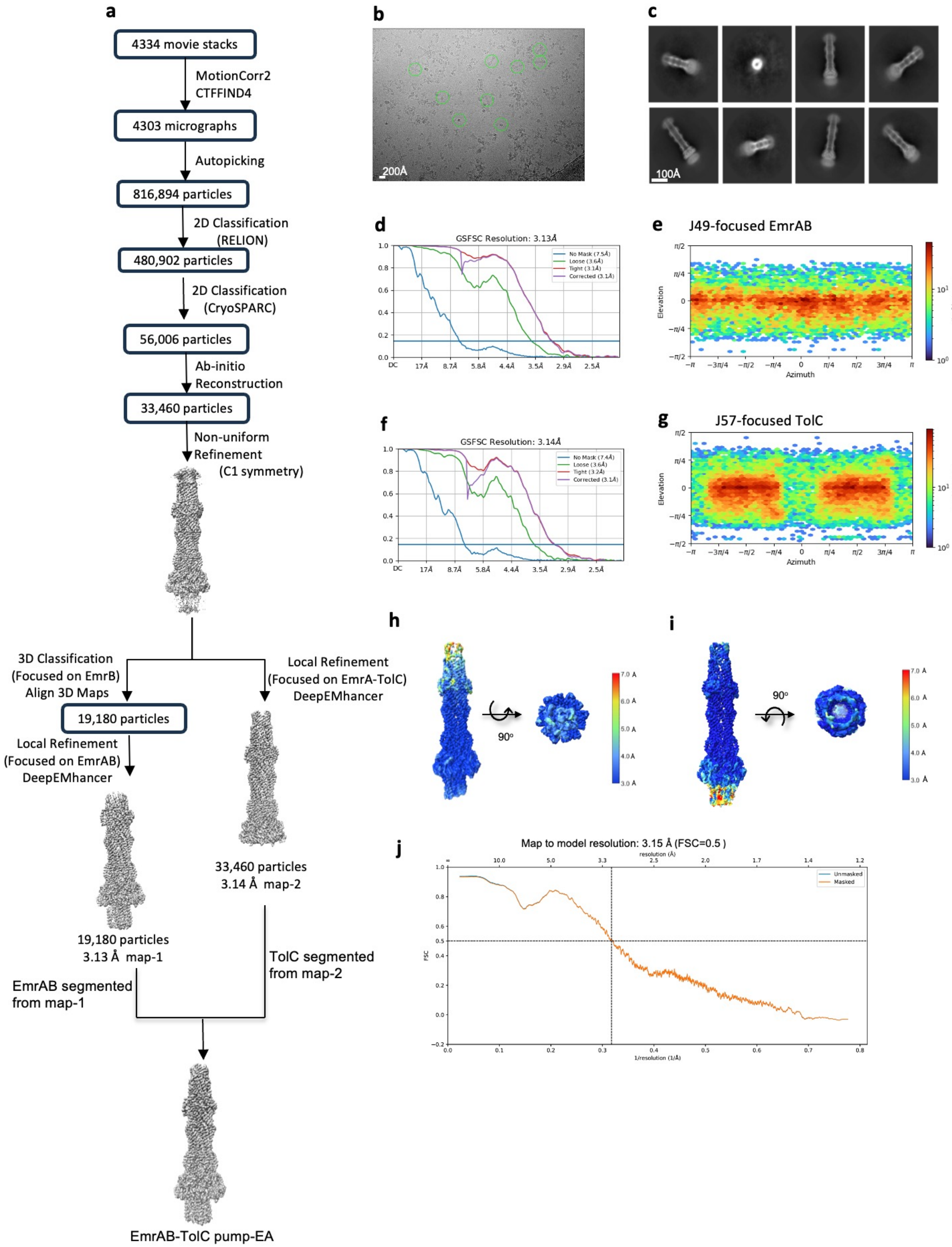
a
4334 movie stacks
MotionCorr2
CTFFIND4
4303 micrographs
Autopicking
816,894 particles
2D Classification
(RELION)
480,902 particles
2D Classification
(CryoSPARC)
56,006 particles
Ab-initio
Reconstruction
33,460 particles
Non-uniform
Refinement
(C1 symmetry)
3D Classification
(Focused on EmrB)
Align 3D Maps
19,180 particles
Local Refinement
(Focused on EmrAB)
DeepEMhancer
19,180 particles
3.13 Å map-1
Local Refinement
(Focused on EmrA-TolC)
DeepEMhancer
33,460 particles
3.14 Å map-2
EmrAB segmented
from map-1
TolC segmented
from map-2
EmrAB-TolC pump-EA
b
200Å
c
100Å
d
GSFSC Resolution: 3.13Å
No Mask (7.5Å)
Loose (3.6Å)
Tight (3.1Å)
Corrected (3.1Å)
e
J49-focused EmrAB
Elevation
Azimuth
# of images
f
GSFSC Resolution: 3.14Å
No Mask (7.4Å)
Loose (3.6Å)
Tight (3.2Å)
Corrected (3.1Å)
g
J57-focused TolC
Elevation
Azimuth
# of images
h
90°
i
90°
j
Map to model resolution: 3.15 Å (FSC=0.5)
resolution (Å)
Unmasked
Masked
FSC
1/resolution (1/Å)

Supplementary Figure 2 | Cryo-EM data analysis of EmrAB-TolC pump-EA. a, Processing workflow of EmrAB-TolC pump-EA. **b,** Representative cryo-EM image of the EmrAB-TolC pump-EA complex. The image has been corrected for drift (scale bar 200 Å). **c,** Typical two-dimensional class averages of the particles (scale bar 100 Å). **d,** CryoSPARC reported Gold-standard Fourier shell correlation (FSC) indicating the resolution of the density map of the EmrAB-TolC pump-EA (Focused on EmrAB). **e,** Particle angular distribution for the final reconstruction of EmrAB-TolC pump-EA (Focused on EmrAB). **f,** CryoSPARC reported Gold-standard Fourier shell correlation (FSC) indicating the resolution of the density map of the EmrAB-TolC pump-EA (Focused on EmrA-TolC). **g,** Particle angular distribution for the final reconstruction of EmrAB-TolC pump-EA (Focused on EmrA-TolC). **h,** Local resolution estimation via ResMap of the pump-EA (Focused on EmrAB). **i,** Local resolution estimation via ResMap of the pump-FA (Focused on EmrA-TolC). All units are in Angstrom (Å). **j,** Map/model correlations of EmrAB-TolC pump-EA. The model was assessed using Pheix Comprehensive Validation [66].

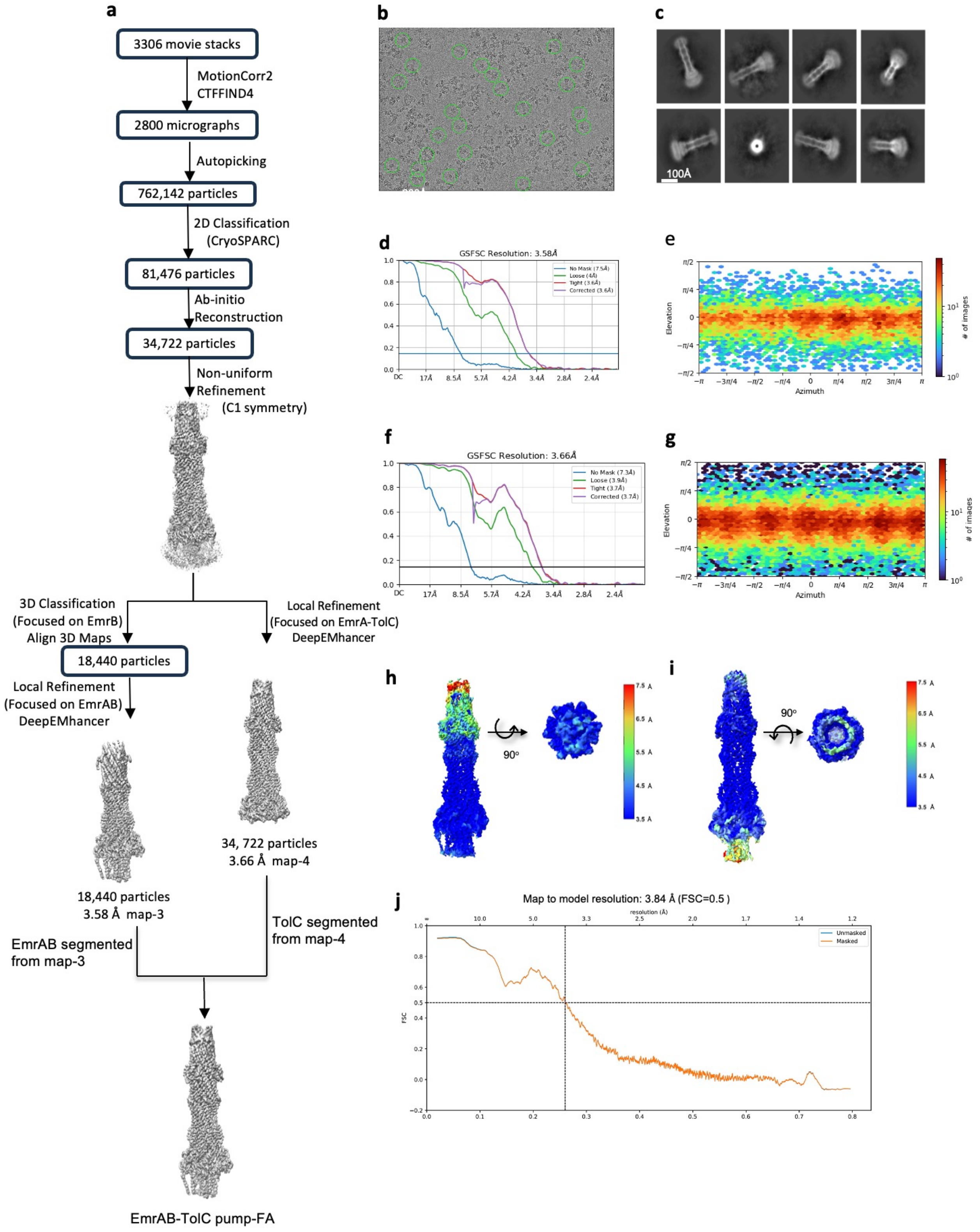
a
3306 movie stacks
MotionCorr2
CTFFIND4
2800 micrographs
Autopicking
762,142 particles
2D Classification
(CryoSPARC)
81,476 particles
Ab-initio
Reconstruction
34,722 particles
Non-uniform
Refinement
(C1 symmetry)
3D Classification
(Focused on EmrB)
Align 3D Maps
18,440 particles
Local Refinement
(Focused on EmrA-TolC)
DeepEMhancer
Local Refinement
(Focused on EmrAB)
DeepEMhancer
34, 722 particles
3.66 Å map-4
18,440 particles
3.58 Å map-3
EmrAB segmented
from map-3
TolC segmented
from map-4
EmrAB-TolC pump-FA
b
c
100Å
d
GSFSC Resolution: 3.58Å
No Mask (7.5Å)
Loose (4Å)
Tight (3.6Å)
Corrected (3.6Å)
e
Elevation
Azimuth
# of images
f
GSFSC Resolution: 3.66Å
No Mask (7.3Å)
Loose (3.9Å)
Tight (3.7Å)
Corrected (3.7Å)
g
Elevation
Azimuth
# of images
h
90°
i
90°
j
Map to model resolution: 3.84 Å (FSC=0.5)
resolution (Å)
FSC
Unmasked
Masked

Supplementary Figure 3 | Cryo-EM data analysis of EmrAB-TolC pump-FA. a, Processing workflow of EmrAB-TolC pump-FA. **b,** Representative cryo-EM image of the EmrAB-TolC pump-FA complex. The image has been corrected for drift (scale bar 200 Å). **c,** Typical two-dimensional class averages of the particles (scale bar 100 Å). **d,** CryoSPARC reported Gold-standard Fourier shell correlation (FSC) indicating the resolution of the density map of the EmrAB-TolC pump-FA (Focused on EmrAB). **e,** Particle angular distribution for the final reconstruction of EmrAB-TolC pump-FA (Focused on EmrAB). **f,** CryoSPARC reported Gold-standard Fourier shell correlation (FSC) indicating the resolution of the density map of the EmrAB-TolC pump-FA (Focused on EmrA-TolC). **g,** Particle angular distribution for the final reconstruction of EmrAB-TolC pump-FA (Focused on EmrA-TolC). **h,** Local resolution estimation via ResMap of the pump-FA (Focused on EmrAB). **i,** Local resolution estimation via ResMap of the pump-FA (Focused on EmrA-TolC). All units are in Angstrom (Å). **j,** Map/model correlations of EmrAB-TolC pump-FA. The model was assessed using Pheix Comprehensive Validation [66].

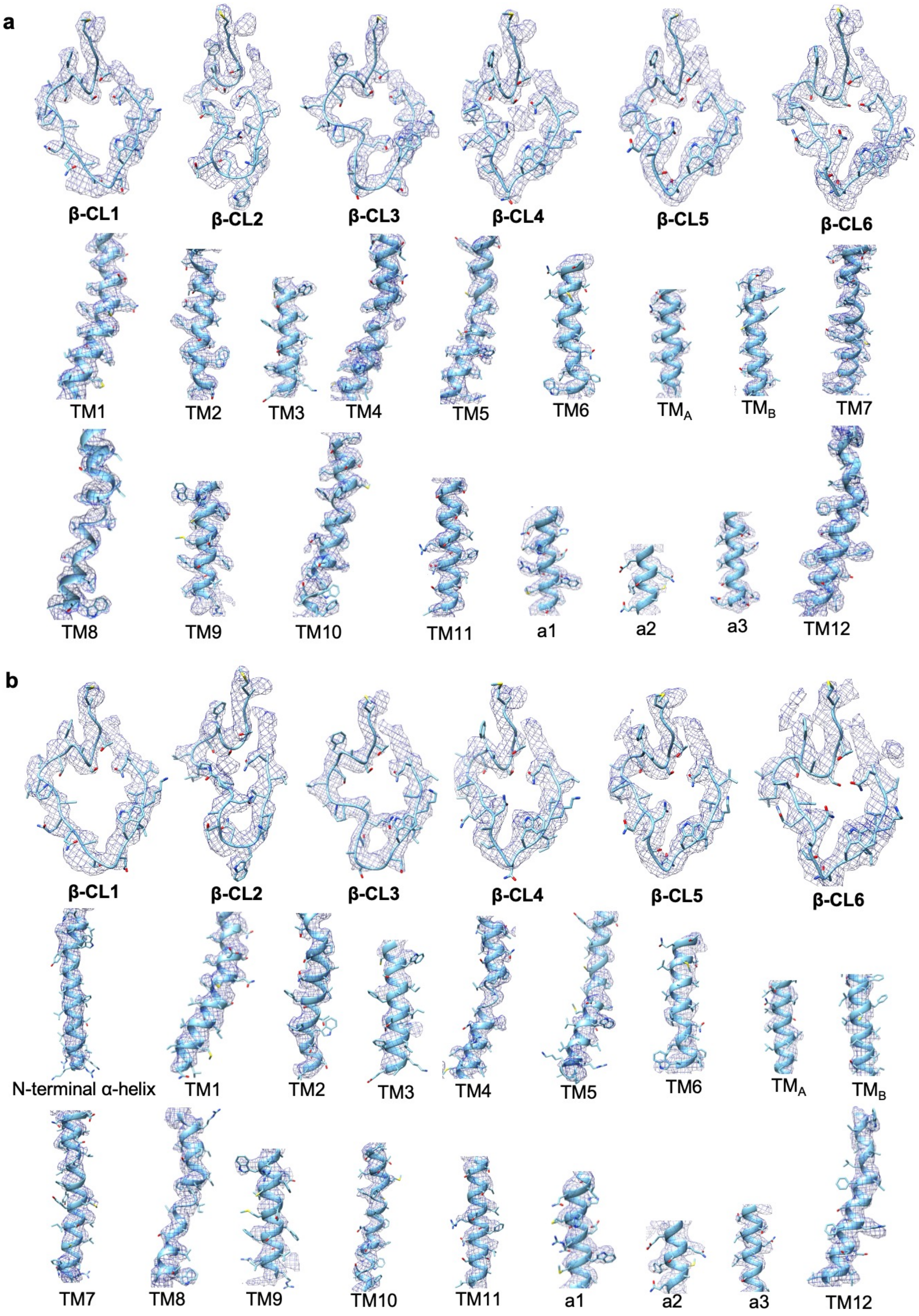

Supplementary Figure 4 | Individual Cryo-EM density maps are displayed for representative secondary structures with their corresponding models. a, Maps and models for EmrAB-TolC pump-EA, including β-CLs of EmrA, and α-helices of EmrB. **b,** Maps and models for EmrAB-TolC pump-FA, including β-CLs and N-terminal α-helix of EmrA, and α-helices of EmrB. Map threshold = 0.15.

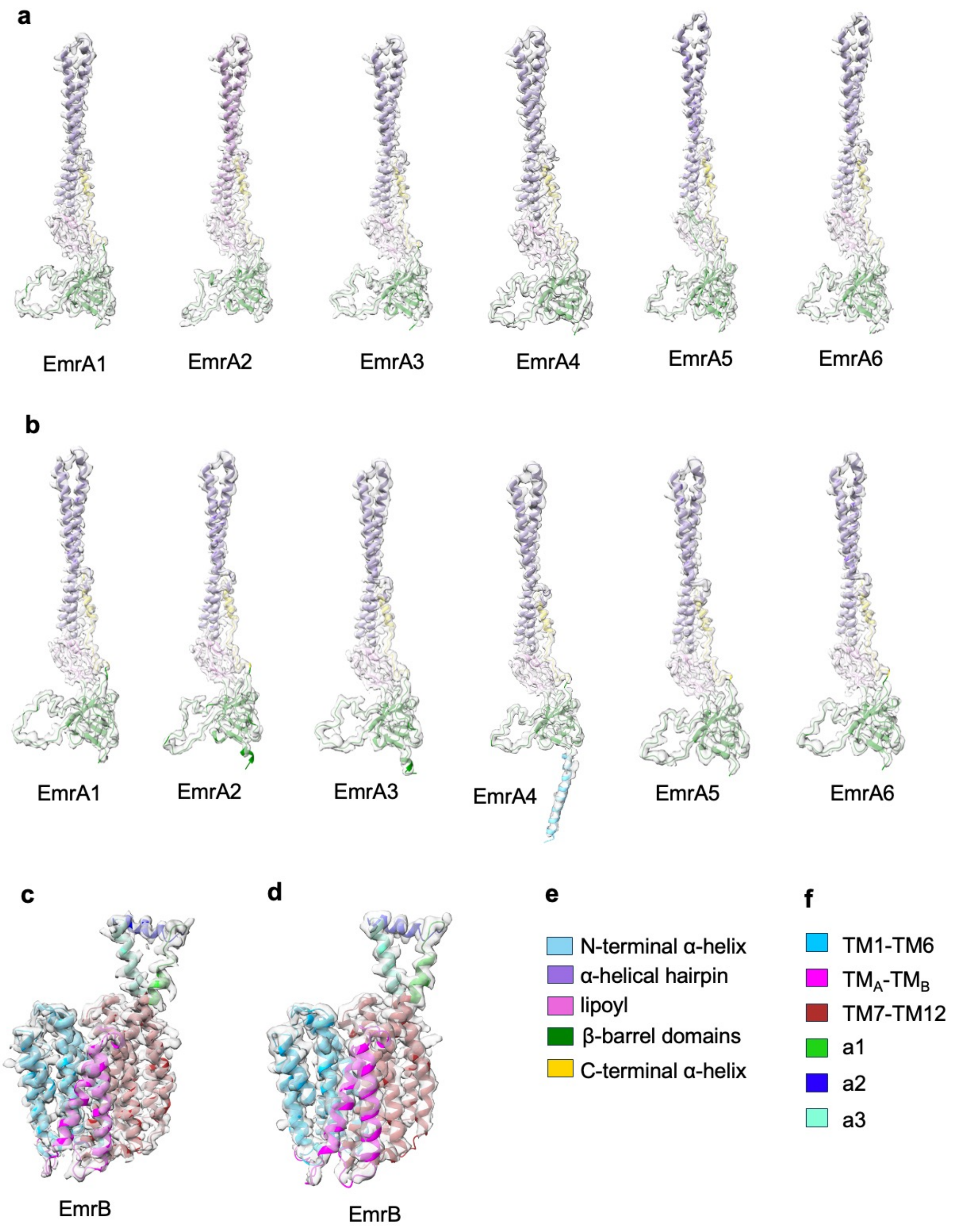

Supplementary Figure 5 | Cryo-EM maps and fitted models of EmrA and EmrB. Cryo-EM maps of segmented protomers are shown: **a,** Maps and models for EmrA1-6 of pump-EA; **b,** Maps and models for EmrA1-6 of pump-FA; **c,** Map and model for EmrB of pump-EA; **d,** Map and model for EmrB of pump-FA. Domains and subdomains of EmrA are color-coded according to panel **e**, while those for EmrB follow the scheme in panel **f**. Map threshold = 0.15.

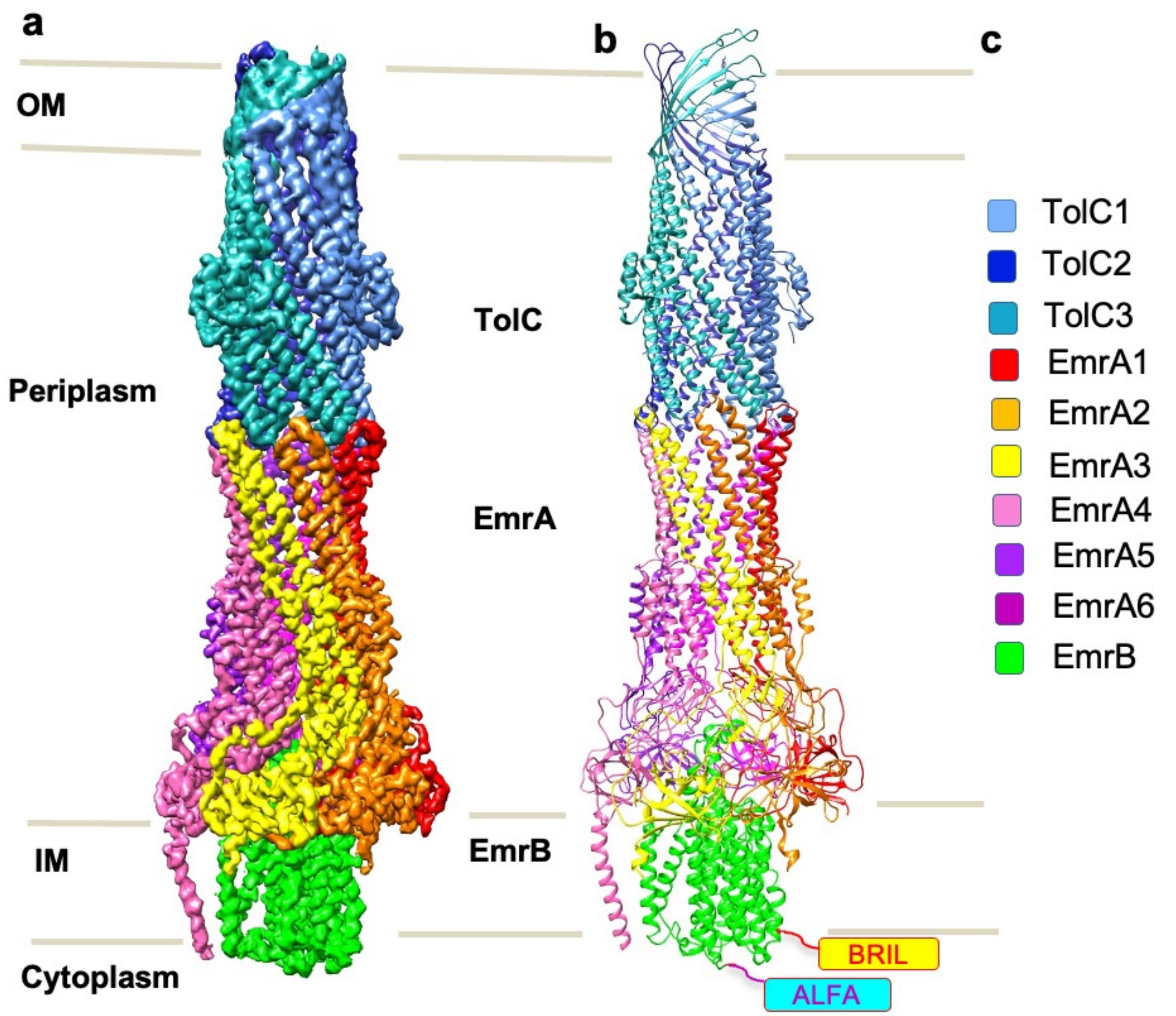

Supplementary Figure 6 | Structure of EmrAB-TolC pump-FA. a, Map of pump-FA. **b,** Ribbon representation of the pump, with the BRIL protein incorporated at the N-terminus and the ALFA-tag fused at the C-terminus of EmrB, respectively. The protomers are color-coded as in **c.** The structure of EmrAB-TolC pump-FA is similar to that of pump-EA, with both having a 3:6:1 TolC:EmrA:EmrB protomer ratio. The map of the N-terminal TM helix of EmrA protomer 4 spans the entire inner membrane (IM), while the densities of the other five N-terminal TM helices are less-were defined.

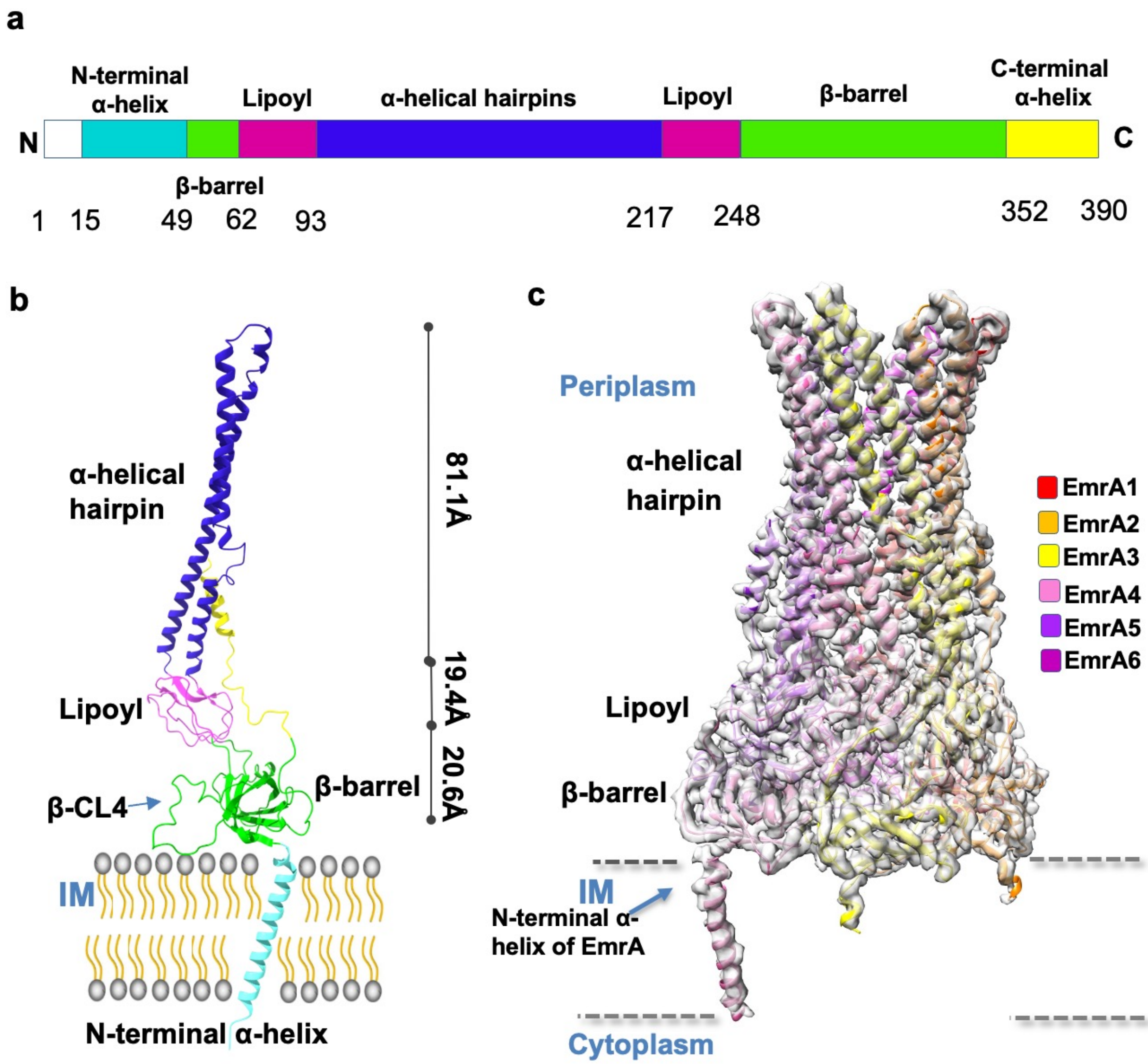

Supplementary Figure 7 | Structure of EmrA in pump FA. a, Linear representation of EmrA (the color code in a is also used for the domains and subdomains in **b**. The numbers below the color-coded bar indicate the approximate positions within the amino acid sequence of EmrA. **b,** Ribbon representation of EmrA protomer 4. **c,** Cryo-EM map of the EmrA hexamer with fitted model (left). Protomers are color-coded accordingly (right). The density map of the N-terminal α-helix of EmrA protomer 4 spans the entire IM, while the density maps of the other five N-terminal TM helices are less well-defined

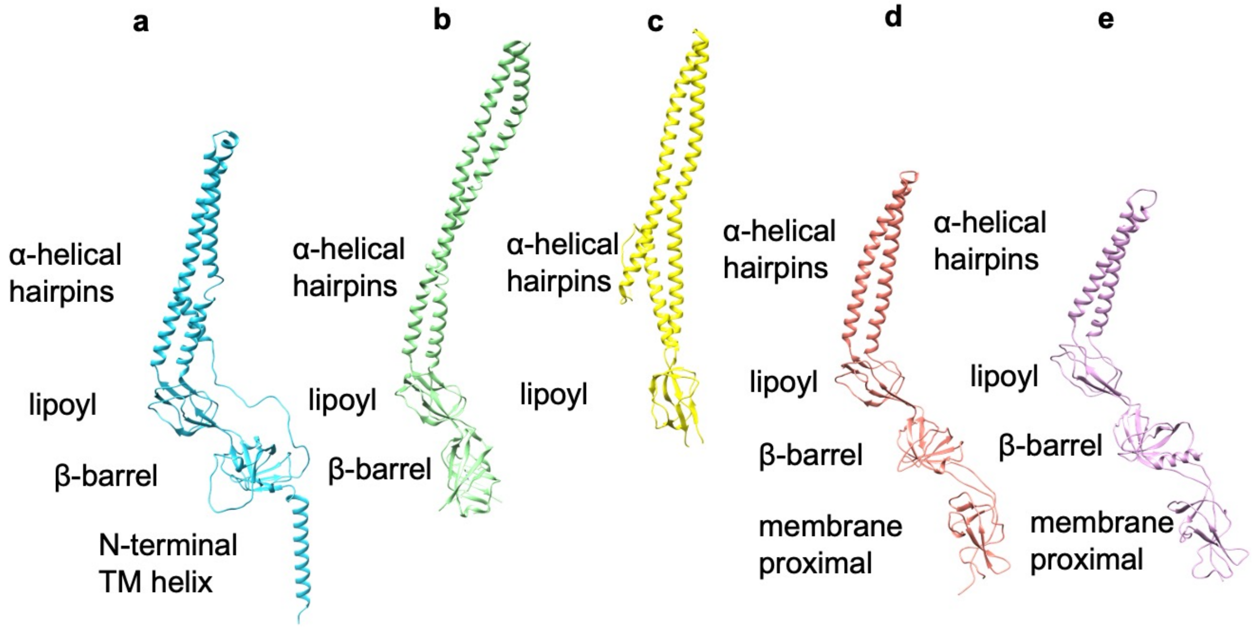

Supplementary Figure 8 | Structural comparison of EmrA, aaEmrA, HlyD, MacA and AcrA. EmrA **a** and its homologue aaEmrA (PDB code: 4TKO) **b** and HlyD (PDB code: 5C21) **c** lack the membrane-proximal domains present in MacA (PDB code: 5NIK) **d** and AcrA (PDB code: 5V5S) **e**, which mediate interactions with periplasmic extensions of the inner membrane transporters MacB and AcrB, respectively.

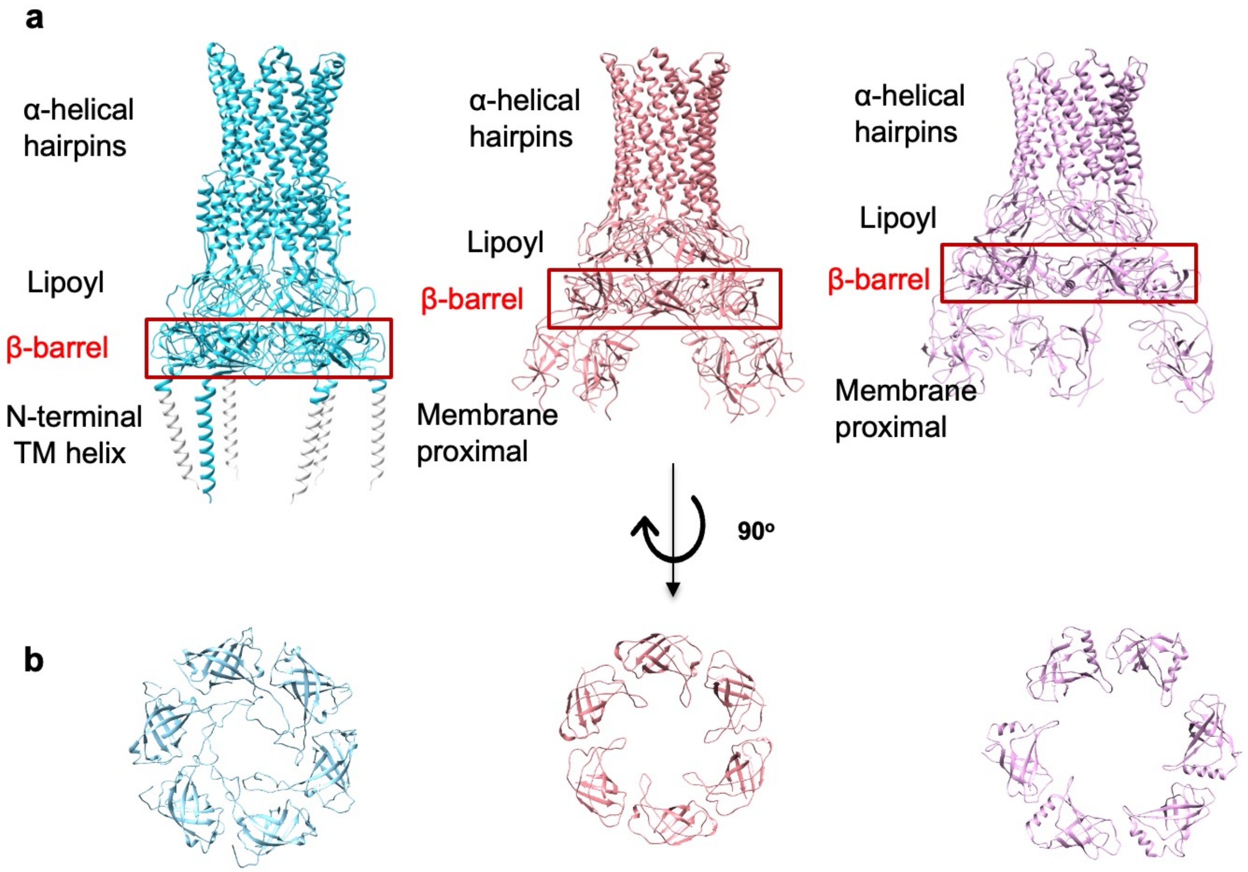

Supplementary Figure 9 | Structural comparison of EmrA, MacA, and AcrA hexamers. a, Side-by-side comparison of EmrA (left), MacA (PDB code: 5NIK; middle), and AcrA (PDB code: 5V5S; right) hexamers. **b,** Comparison of the β-barrel domains of EmrA (left), MacA (middle), and AcrA (right) hexamers. The conserved β-CLs of EmrA adopt various conformations, while the equivalent parts of MacA and AcrA are short and adopt similar conformations.

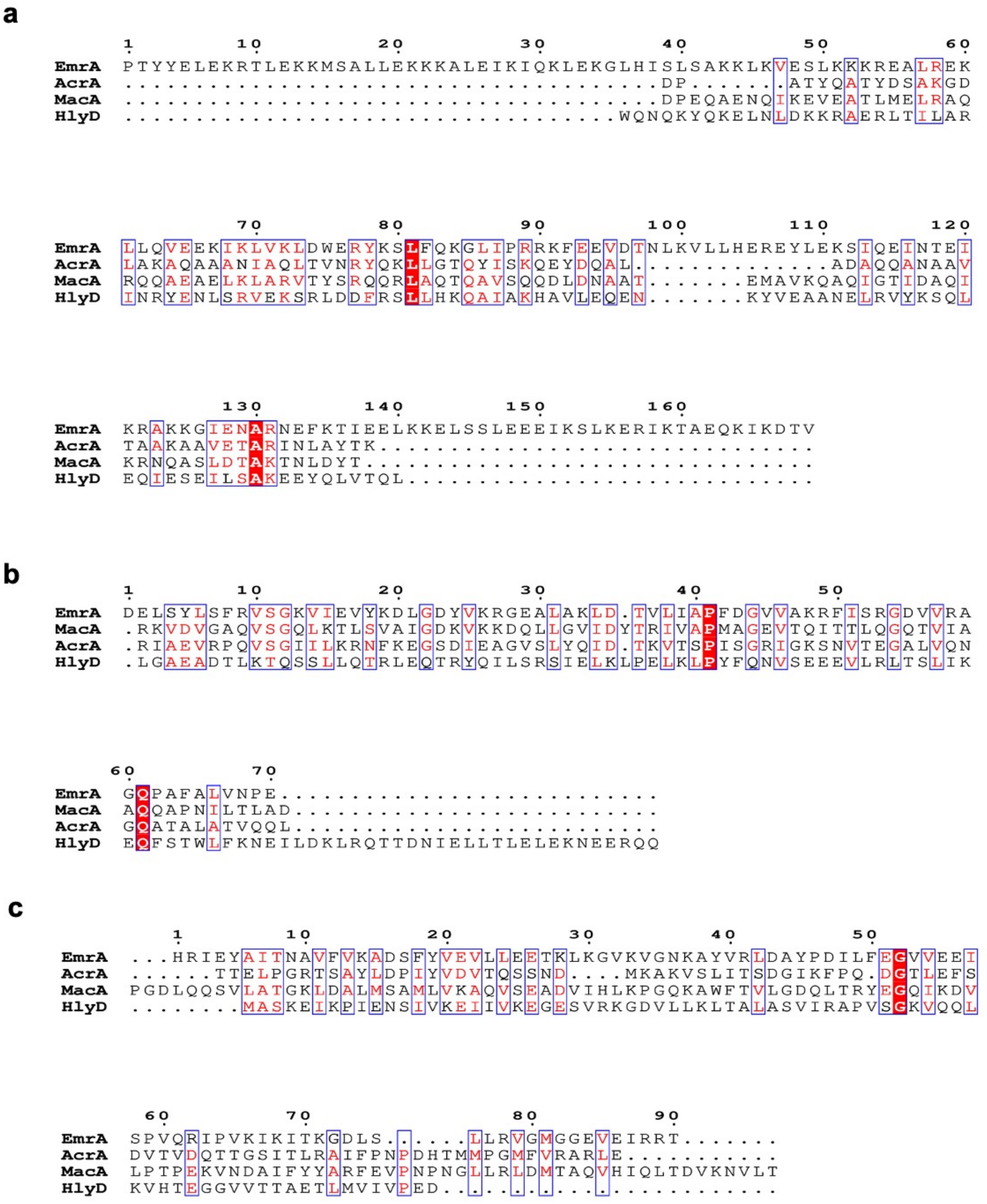

Supplementary Figure 10 | Sequence alignments between EmrA, AcrA, MacA and HlyD. a, Sequence alignment for α-helical hairpin domains. **b,** Sequence alignment for lipoyl domains. **c,** Sequence alignment for β-barrel domains.

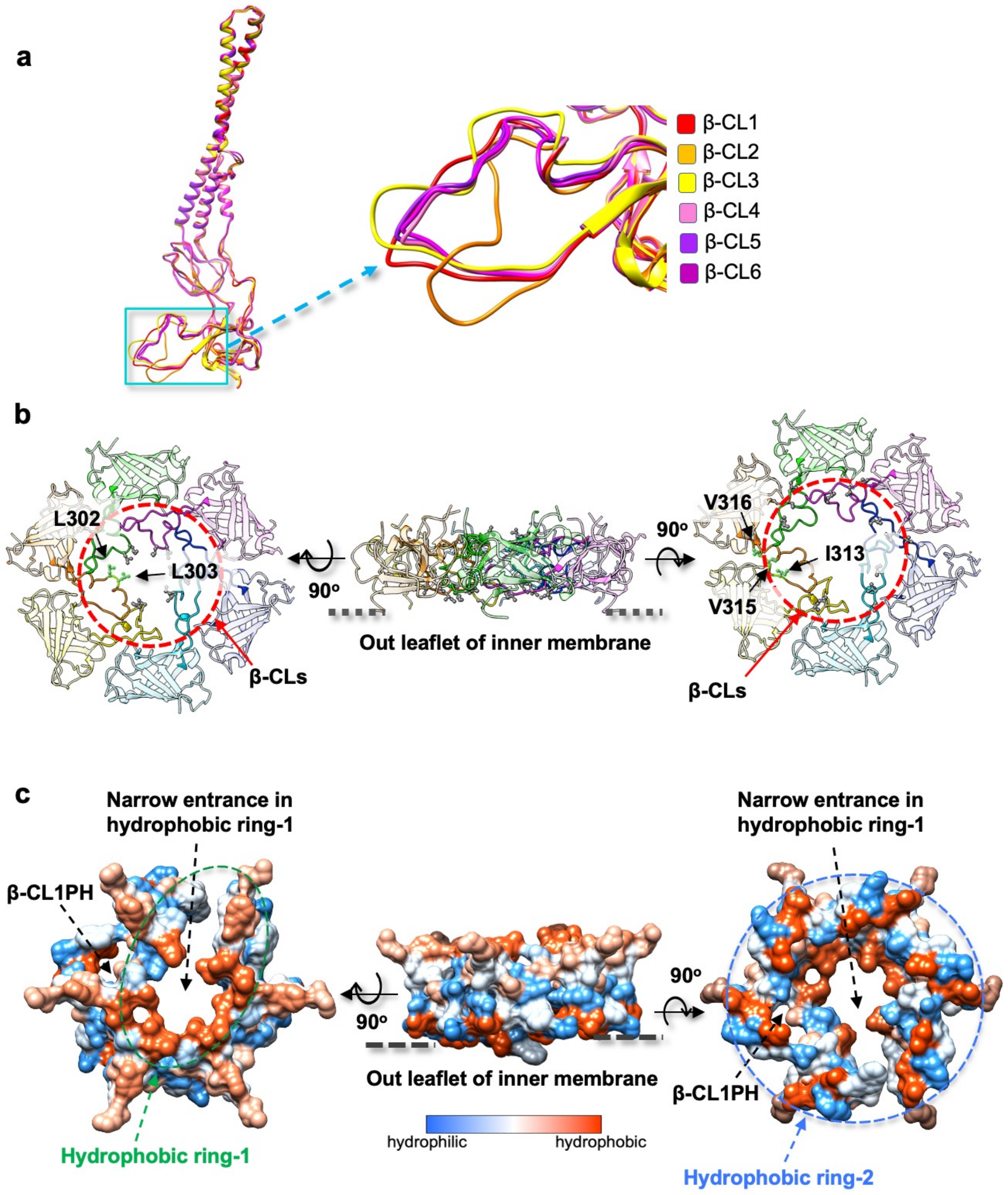

Supplementary Figure 11 | Structural characteristics of β-CL region. a, Superposition of six protomers of EmrA (left) and enlargement of the boxed region (middle) showing various conformations of β-CL1 to β-CL6. β-CLs are color-coded accordingly (right). **b,** The IM distal and proximal sides (left and right, viewed perpendicular to the membrane) and side view (middle, viewed parallel to the membrane) of EmrA β-barrel domains showing the β-CL region and two hydrophobic rings. **c,** Surface representations of β-CL region (with the same views as in **B**). Residues are coloured based on hydrophobicity.

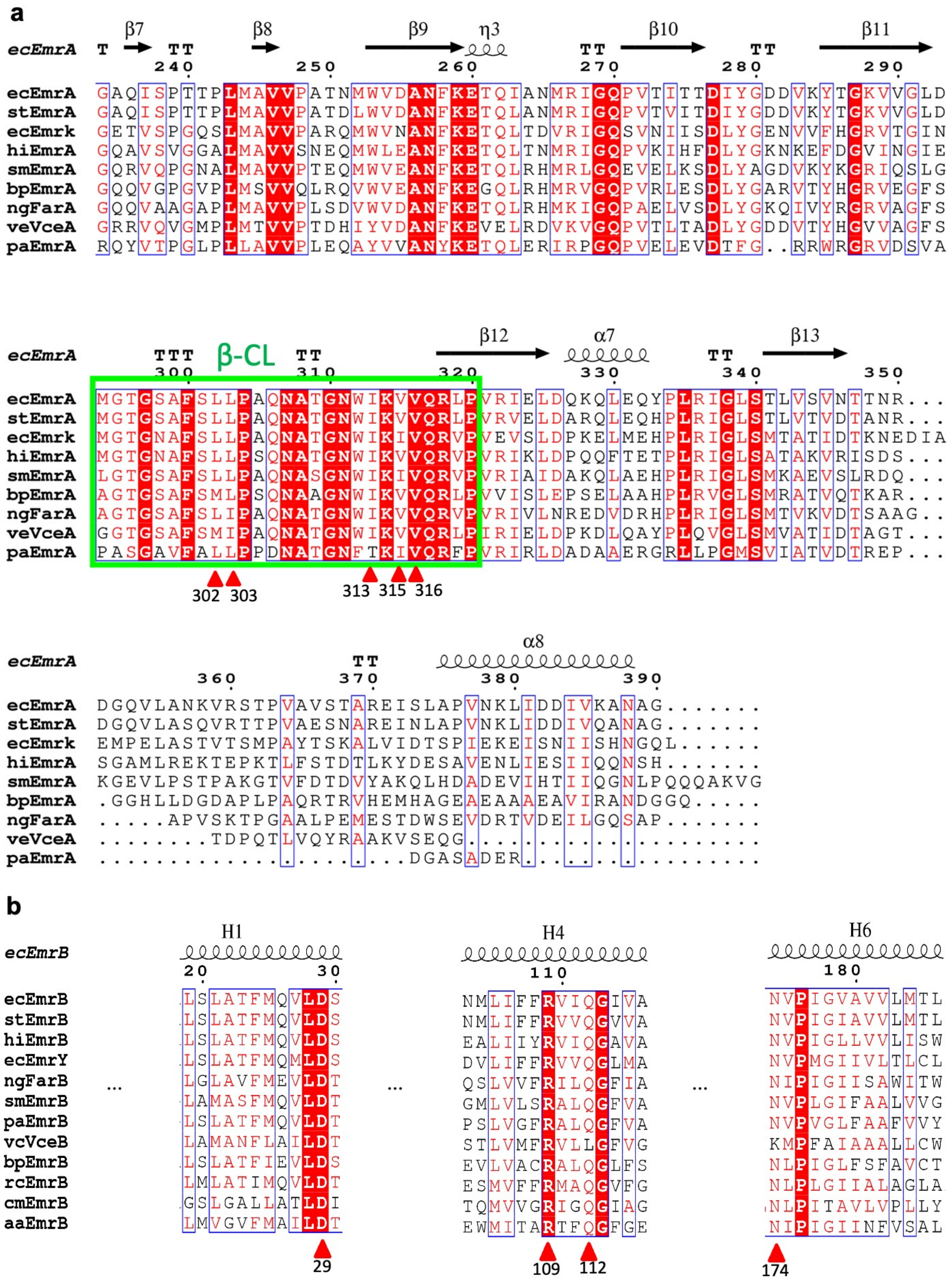
a
ecEmrA
β7
β8
β9
η3
β10
β11
β12
α7
β13
α8
β-CL
ecEmrA
stEmrA
ecEmrk
hiEmrA
smEmrA
bpEmrA
ngFarA
veVceA
paEmrA
302
303
313
315
316
b
ecEmrB
H1
H4
H6
ecEmrB
stEmrB
hiEmrB
ecEmrY
ngFarB
smEmrB
paEmrB
vcVceB
bpEmrB
rcEmrB
cmEmrB
aaEmrB
29
109
112
174

Supplementary Figure 12 | Sequence alignments of EmrA and EmrB with their homologues, respectively. a, The residues L302, L303, I313, V315 and V316 in the β-CL (green box) of β-barrel domain are conserved among EmrA homologues. The sequences used were from *Escherichia coli* (ecEmrA, P27303; ecEmrK, P52599), *Salmonella Typhimurium* (stEmrA, E8XK58), *Haemophilus influenza* (hiEmrA, P44928), *Stenotrophomonas maltophilia* (smEmrA, A0A7V8FFT6), *Burkholderia pseudomallei* (bpEmrA, Q3JLM5), *Neisseria gonorrhoeae* (ngFarA, Q9RQ30), *Vibrio cholerae* (vcVceA, O51918), *Pseudomonas aeruginosa* (paEmrA, A0A3M5E5C8), with corresponding UniProt codes. **b,** The residues D29, R109 and Q112 are conserved among EmrB homologues. The sequences used were from *Escherichia coli* (ecEmrB, P0AEJ0; ertEmrY, P52600), *Aquifex aeolicus* (aaEmrB, O67160), *Vibrio cholerae* (vcVceB, A0A5C9SY76), *Stenotrophomonas maltophilia* (smEmrB, A0A0D0JC09), *Rhodobacter capsulatus* (rcEmrB, D5ASL8), *Salmonella Typhimurium* (stEmrB, A0A731QJR1), *Pseudomonas aeruginosa* (paEmrB, A0A0N7HJZ5), *Cupriavidus metallidurans* (cmEmrB, A0A132HJX6), *Burkholderia pseudomallei* (bpEmrB, Q3JSY6), *Haemophilus influenza* (hiEmrB, A0A377IYF3) and *Neisseria gonorrhoeae* (ngFarB, Q9RQ29), with corresponding UniProt codes. Alignments were performed over the whole sequences, but for clarity, only the relevant regions are shown. Residues that are strictly conserved are highlighted with white text on a red background, while highly conserved residues are highlighted with red text on a white background. The secondary structure is marked above.

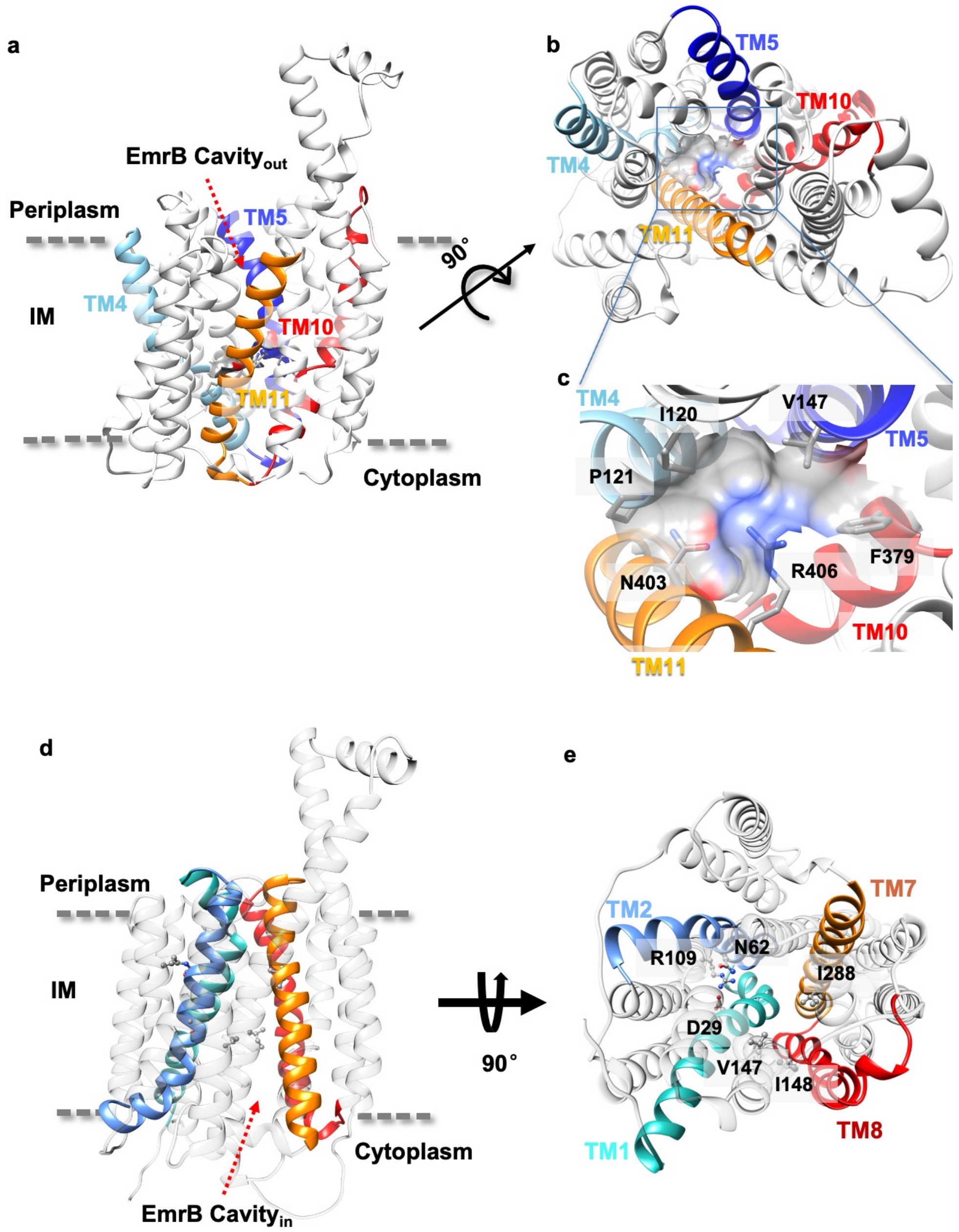

Supplementary Figure 13 | The central aqueous cavities and gating helices of EmrB. a, b, Side and periplasmic views of the EmrB structure in the outward-open state. Helices TM4 and TM5 in the N-domain, along with TM10 and TM11 in the C-domain, constitute a cytoplasmic gate. **c,** Stick and surface representations of residues I120 and P121 in TM4, V147 in TM5, F379 in TM10, and N403 and R406 in TM11, demonstrate interactions sealing the bottom of cavity$_{out}$ (periplasmic view). **d,** e, Side and cytoplasmic views of the homology model of EmrB in the inward-open state showing cavity$_{in}$. Interactions of side chains between helices TM1 and TM2 in the N-domain, and TM7 and TM8 in the C-domain form a periplasmic gate.

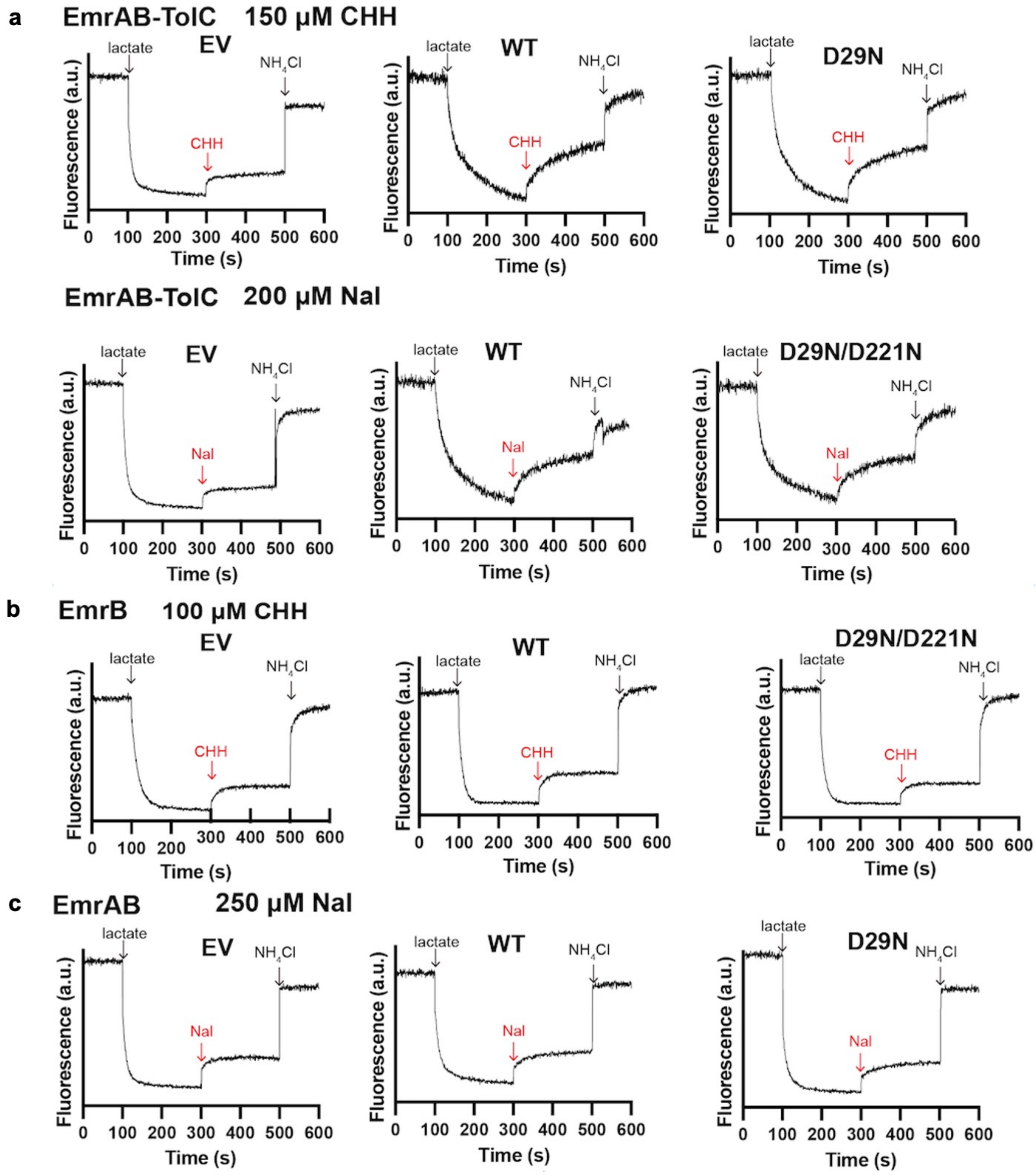
a
EmrAB-TolC 150 µM CHH
EV
WT
D29N
lactate
CHH
NH4Cl
Fluorescence (a.u.)
Time (s)
0 100 200 300 400 500 600
EmrAB-TolC 200 µM NaI
EV
WT
D29N/D221N
NaI
b
EmrB 100 µM CHH
EV
WT
D29N/D221N
c
EmrAB 250 µM NaI
EV
WT
D29N

Supplementary Figure 14 | Proton transport by everted membrane vesicles containing EmrB, EmrAB or EmrAB-TolC. The transmembrane pH was followed by measuring ACMA fluorescence. Lactate (added after 0.5 min) and NH_4Cl (after 5 min) were used to generate or dissipate pH, respectively. 2-chlorophenylhydrazine hydrochloride (CHH) or nalidixic acid (Nal) was added after 3 min. **a,** Everted membrane vesicles were prepared from *E. coli* C43 (DE3) Δ*acrAB* cells expressing the EmrAB–TolC efflux pumps containing wild-type EmrB, $EmrB_{D29N}$ and $EmrB_{D29N/D211N}$, respectively. CHH (150 μM) or Nal (200 μM) was added to 2 mL of vesicle suspension containing 200 μg total membrane protein. **b,** Everted membrane vesicles were prepared from *E. coli* C43 (DE3) Δ*acrAB* cells expressing the wild-type EmrB and $EmrB_{D29N/D211N}$, respectively. CHH (100 μM) was added to 2 mL of vesicle suspension containing 200 μg total membrane protein. **c,** Everted membrane vesicles were prepared from *E. coli* C43 (DE3) Δ*acrAB* cells expressing the EmrAB efflux pumps containing wild-type EmrB and $EmrB_{D29N}$, respectively. Nal (250 μM) was added to 2 mL of vesicle suspension containing 200 μg total membrane protein. EV: empty-vector controls; WT: wild-type EmrAB-TolC complex.

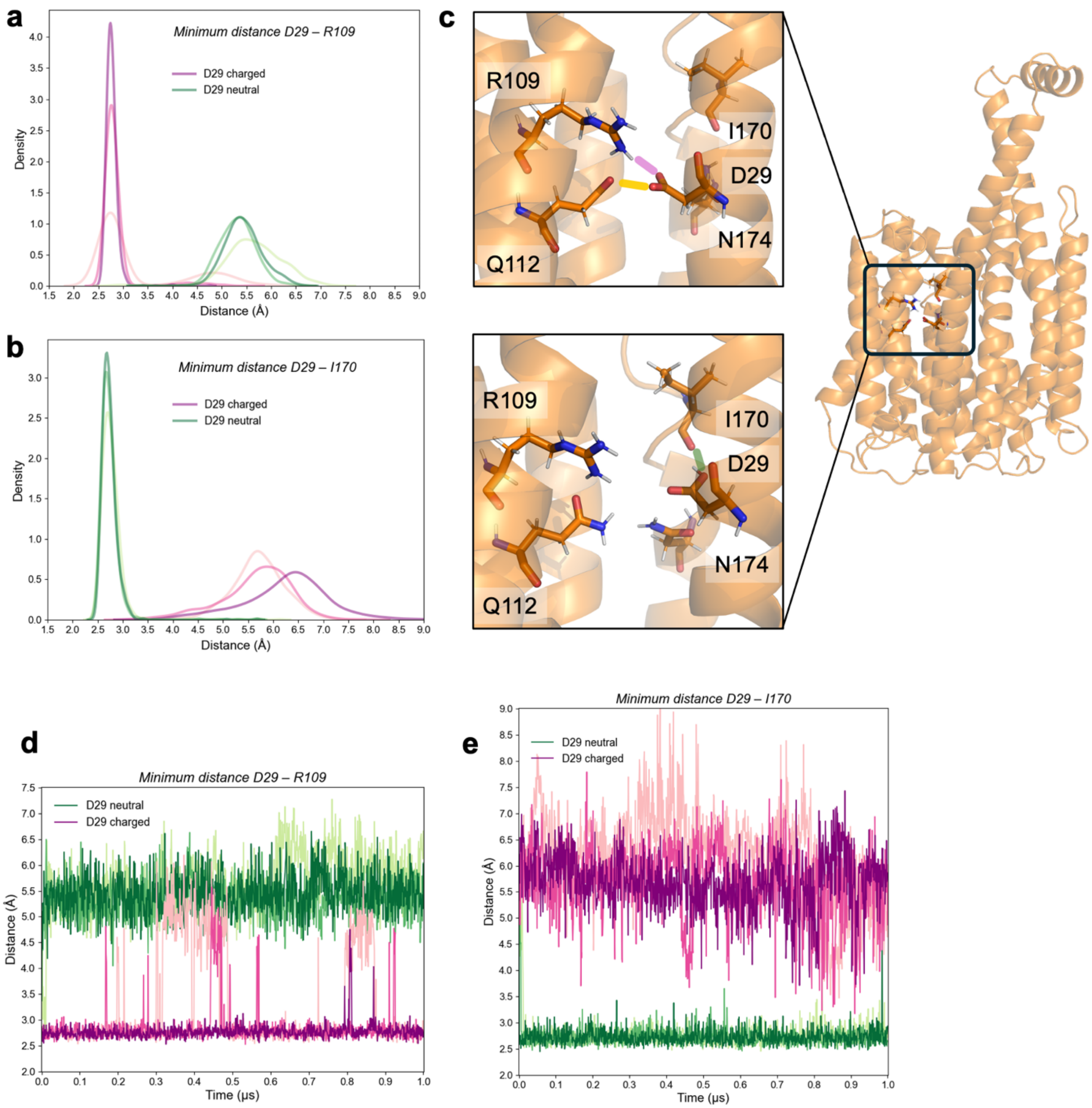

Supplementary Figure 15 | Protonation of D29 alters its local H-bonding network. **a,** Probability distribution of the minimal distance between the side chains of D29 and R109 (heavy atoms) in the charged state (magenta shades) and neutral state (green shades) of D29. **b,** Probability distribution of the minimal distance between the side chain of D29 and the backbone carbonyl group of I170 (heavy atoms) in the charged state (magenta shades) and neutral state (green shades) of D29. D29 reorientates to form a tight H-bond with the backbone of I170 upon protonation. **c,** shows close-up views of the reorientation of D29 from R109 (top left) to I170 (bottom left) upon protonation; the location of the H-bond network around D29 in EmrB is shown on the right. **d**, Minimum distance between D29 and I170. **e**, Minimum distance between D29 and R109. The conformational changes induced by the change from charged to neutral protonation state of D29 occur on a very fast timescale of few ns (lines in green shades) and rapidly converge to a stable state thereafter in each replicate simulation. The change in distance distributions is highly significant in both cases with a p-value < 0.001.

a

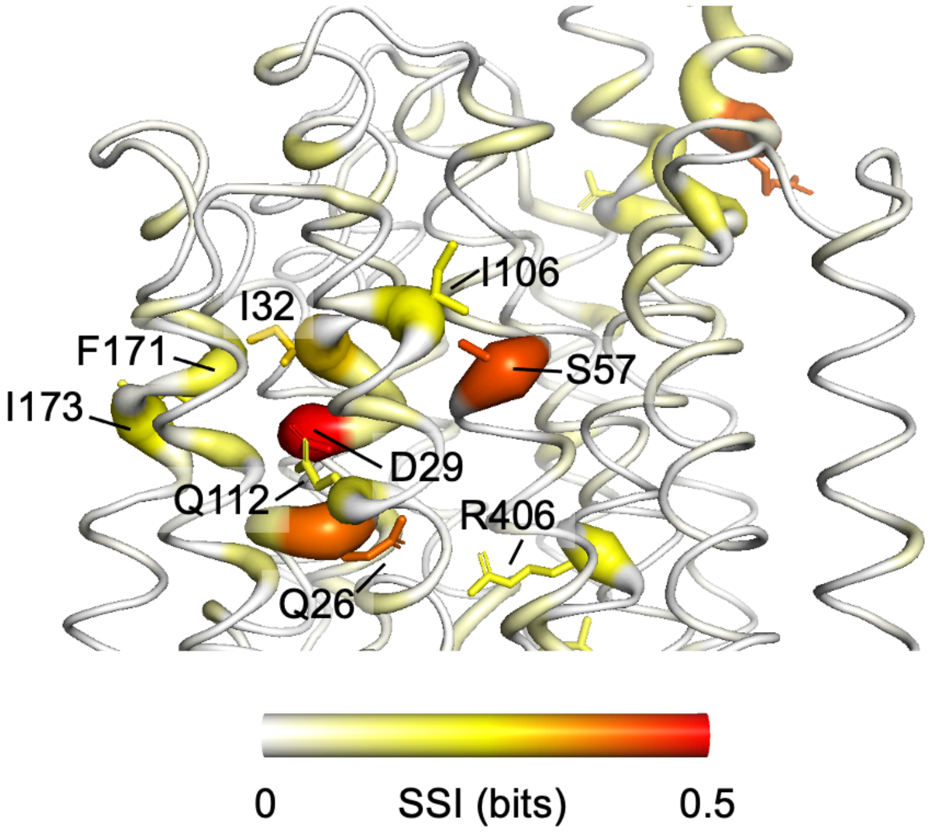

b

Simulation box details

Simulation box dimensions (post-equilibration)	x, y: 100.6Å; z: 131.1Å
Total number of atoms	126439
Number of water molecules	27790
Salt concentration	150mM KCl
Lipid type and composition	POPE 210 : POPG 70

Supplementary Figure 16 | A mutual information analysis reveals medium-to-long range impacts of D29 protonation. a, State-specific information (SSI) (Vögele et al., 2025) highlights residues conformation which are affected by the protonation change of D29 in a colour code from white (least affected) via yellow to red (most affected). The local side chain reorientation of D29 is correlated with conformational changes up to ~16 Å away (R406). **b**, Table of simulation details used for triplicate simulations under both conditions.

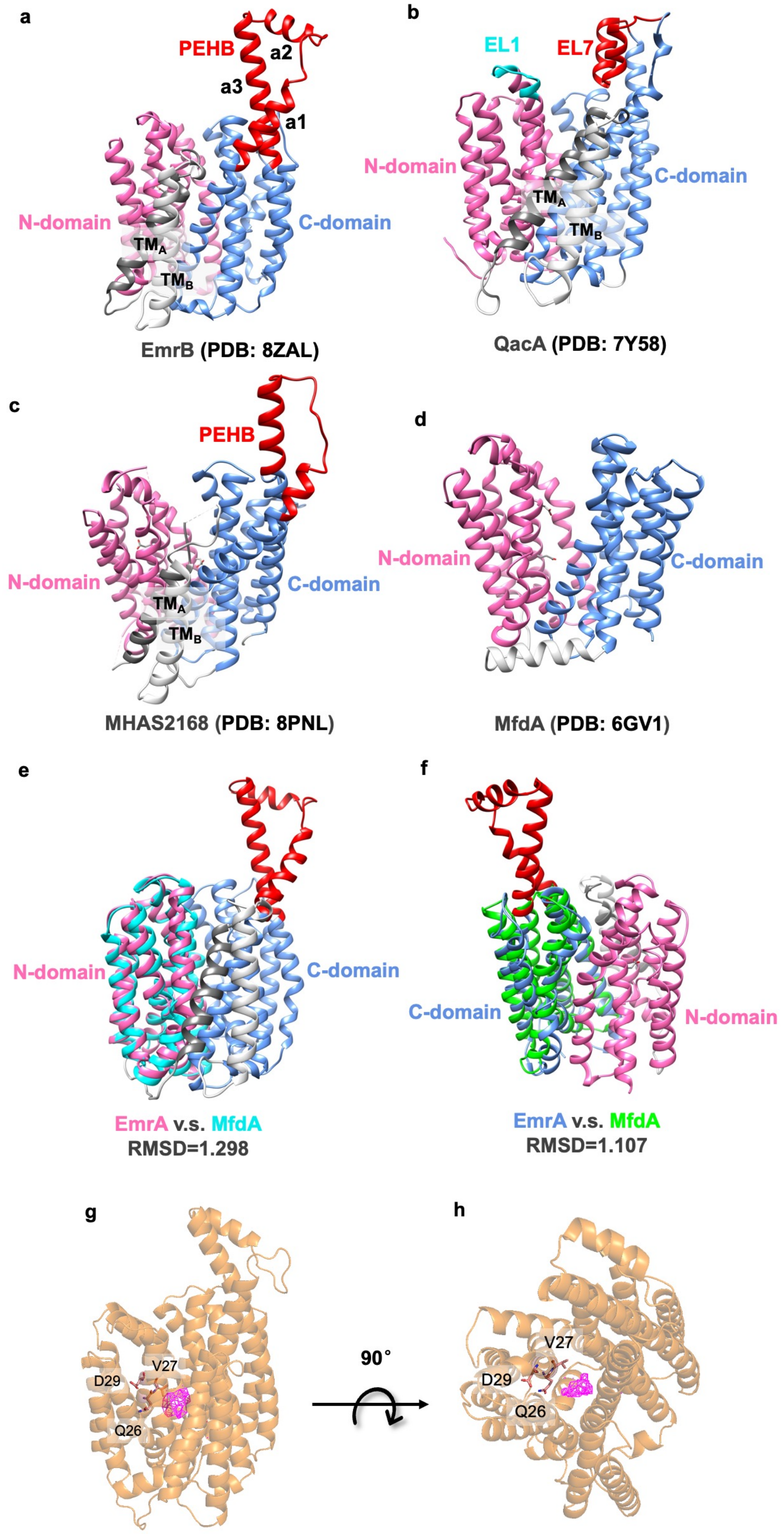
a
PEHB
a2
a3
a1
N-domain
C-domain
TM$_A$
TM$_B$
EmrB (PDB: 8ZAL)
b
EL1
EL7
N-domain
C-domain
TM$_A$
TM$_B$
QacA (PDB: 7Y58)
c
PEHB
N-domain
C-domain
TM$_A$
TM$_B$
MHAS2168 (PDB: 8PNL)
d
N-domain
C-domain
MfdA (PDB: 6GV1)
e
N-domain
C-domain
EmrA v.s. MfdA
RMSD=1.298
f
C-domain
N-domain
EmrA v.s. MfdA
RMSD=1.107
g
D29
V27
Q26
90°
h
V27
D29
Q26

Supplementary Figure 17 | Comparison between EmrB, QacA, MHAS2168 and MfdA. a-d, Ribbon representations of EmrB, QacA, MHAS2168 and MfdA, respectively. **e,** Superposition of N-domains of EmrB and MfdA. **f,** Superposition of C-domains of EmrB and MfdA. **g, h,** The main density of ensemble-docked poses of the EmrB substrates nalidixic acid and nitroxoline to the outward-open state of EmrB locates to residues Q26 and V27 near D29 (**g:** side view; **h:** top view). The substrate density is shown as magenta mesh. The N- and C-domain structures of EmrB are similar to that of MfdA, which facilitates the identification of residues in EmrB involved in drug resistance.

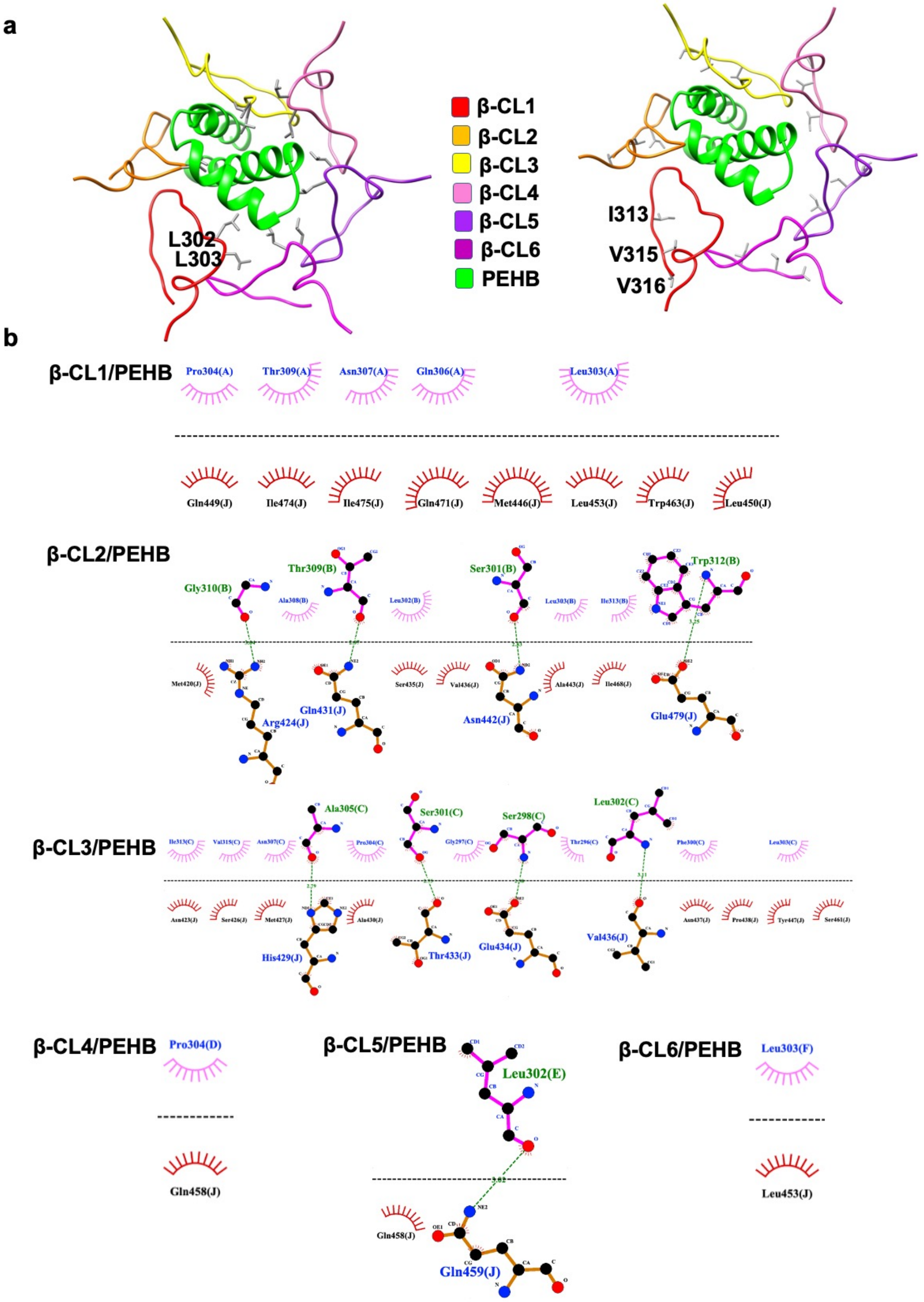

Supplementary Figure 18 | Interactions between β-CLs of EmrA and PEHB of EmrB. **a,** Ribbon representations of β-CLs/PEHB viewed perpendicular to the membrane on the periplasmic side. The residues on hydrophobic ring-1 (L302 and L303, left) and ring-2 (I313, V315 and V316, right) are shown as sticks. The β-CLs and PEHB are color-coded accordingly (middle). **b,** A plot showing contacts between β-CLs and PEHB, respectively. Labels 'A' to 'F' in parentheses following residue numbers denote EmrA1 to EmrA6, respectively; labels 'J' denotes EmrB.

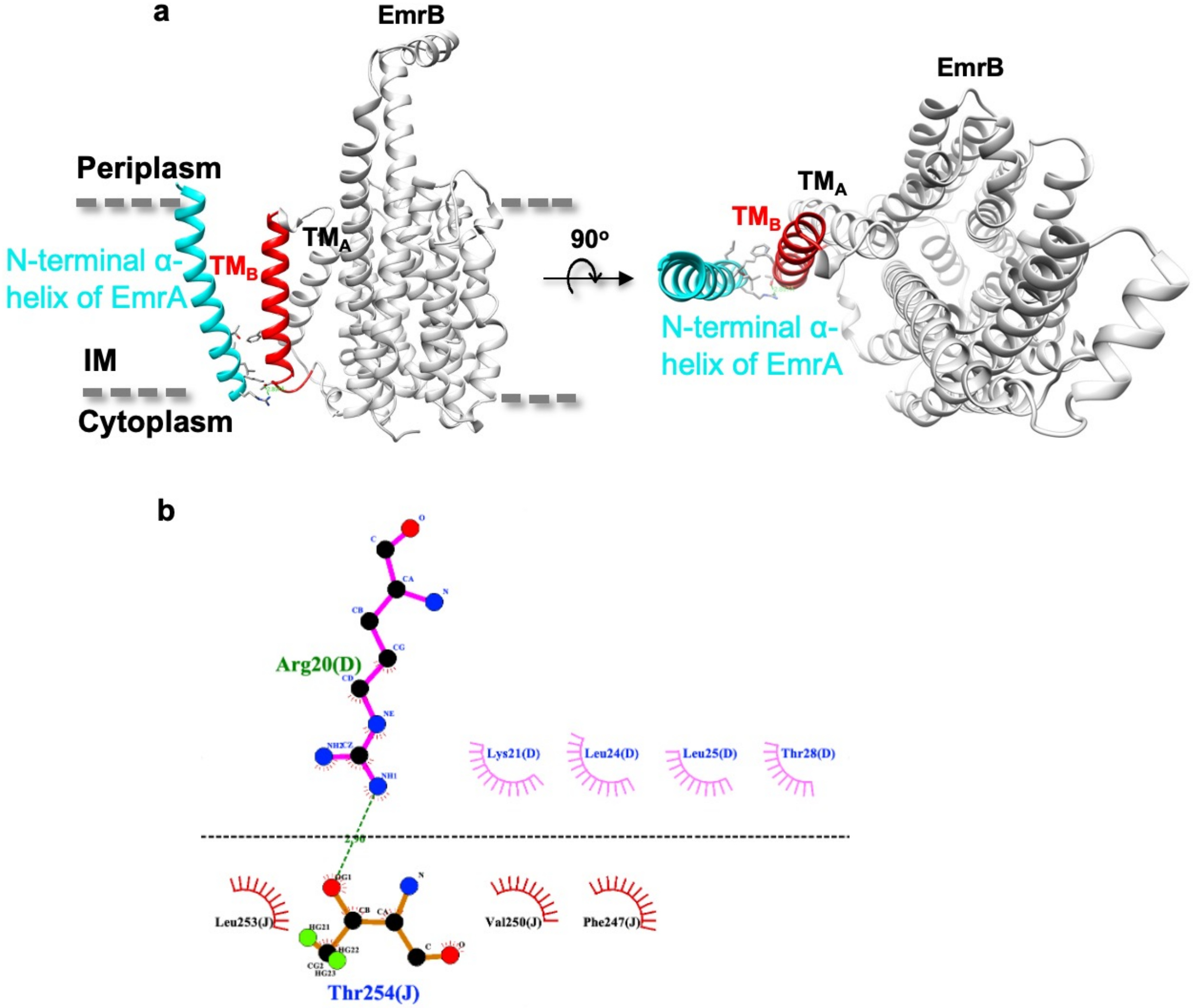

Supplementary Figure 19 l Interactions between the N-terminal a-helix of EmrA and helix TM$_B$ of EmB in the EmrAB-TolC pump-FA. a, Ribbon representations of the N-terminal a-helix of EmrA and EmrB viewed perpendicular (left) and parallel to the membrane on the periplasmic side (righr), respectively. The residues involved in contacts are shown as sticks. The N-terminal a-helix of EmA is colored in cyan, and the helix Hg of EmB is colored in red. **b,** A plot showing contacts between the N-terminal a-helix of EmrA and helix Hg of EmrB. Labels 'D' and 'J' in parentheses following residue numbers denote EmrA4 and EmrB, respectively.

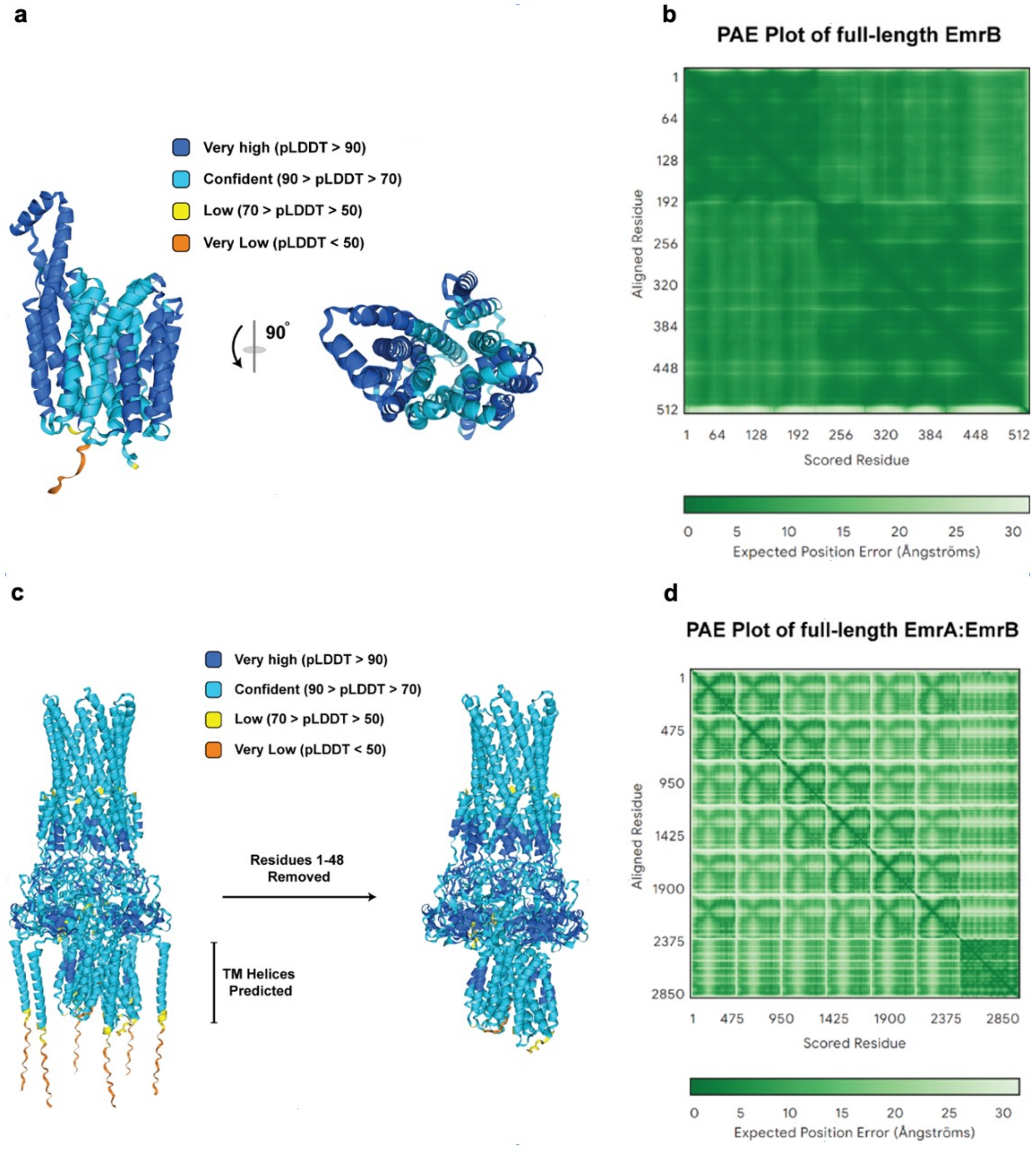

Supplementary Figure 20 | AlphaFold3 Predictions of EmrB and EmrA:EmrB. a, AlphaFold3 predicts EmrB in its outward-open state with high confidence, as demonstrated by the high per-residue confidence throughout the structure. **b,** The predicted aligned error (PAE) plot demonstrates global confidence in the EmrB prediction. **c,** EmrB is predicted in its inward-open state with high confidence when predicted with six molecules of EmrA. The transmembrane helices of EmrA are omitted for easier visualization. **d,** The predicted aligned error (PAE) plot of full-length EmrA:EmrB demonstrates global confidence in the position of individual monomers, as supported by the low-expected position error throughout.

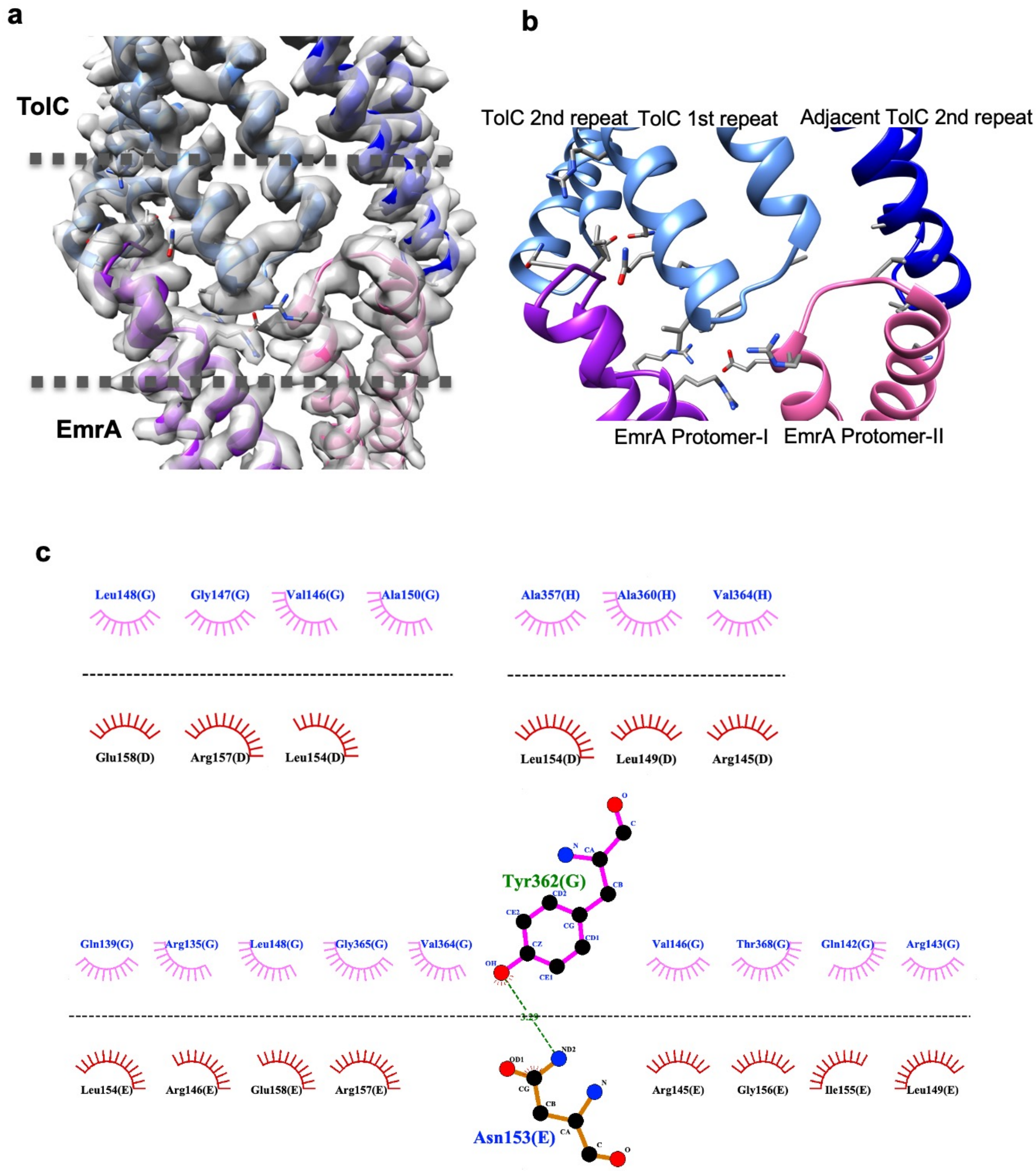

Supplementary Figure 21 | Interfacial contacts between EmrA and TolC. a, Portions of the cryo-EM density map of the pump, combined with the fitted model, showing tip-to-tip interactions between TolC protomers (blue and light blue) and two α-helical hairpins of EmrA (purple and pink). **b,** Closer view of the area between the dashed lines in **a** showing the interaction interfaces between EmrA and TolC. The side chains of the residues participating in contacts are highlighted in stick representation. **c,** A plot showing contacts between EmrA and TolC. Labels 'D' and 'E' in parentheses following residue numbers denote EmrA4 and EmrA5, respectively; labels 'G' and 'H' denote TolC1 and TolC2.

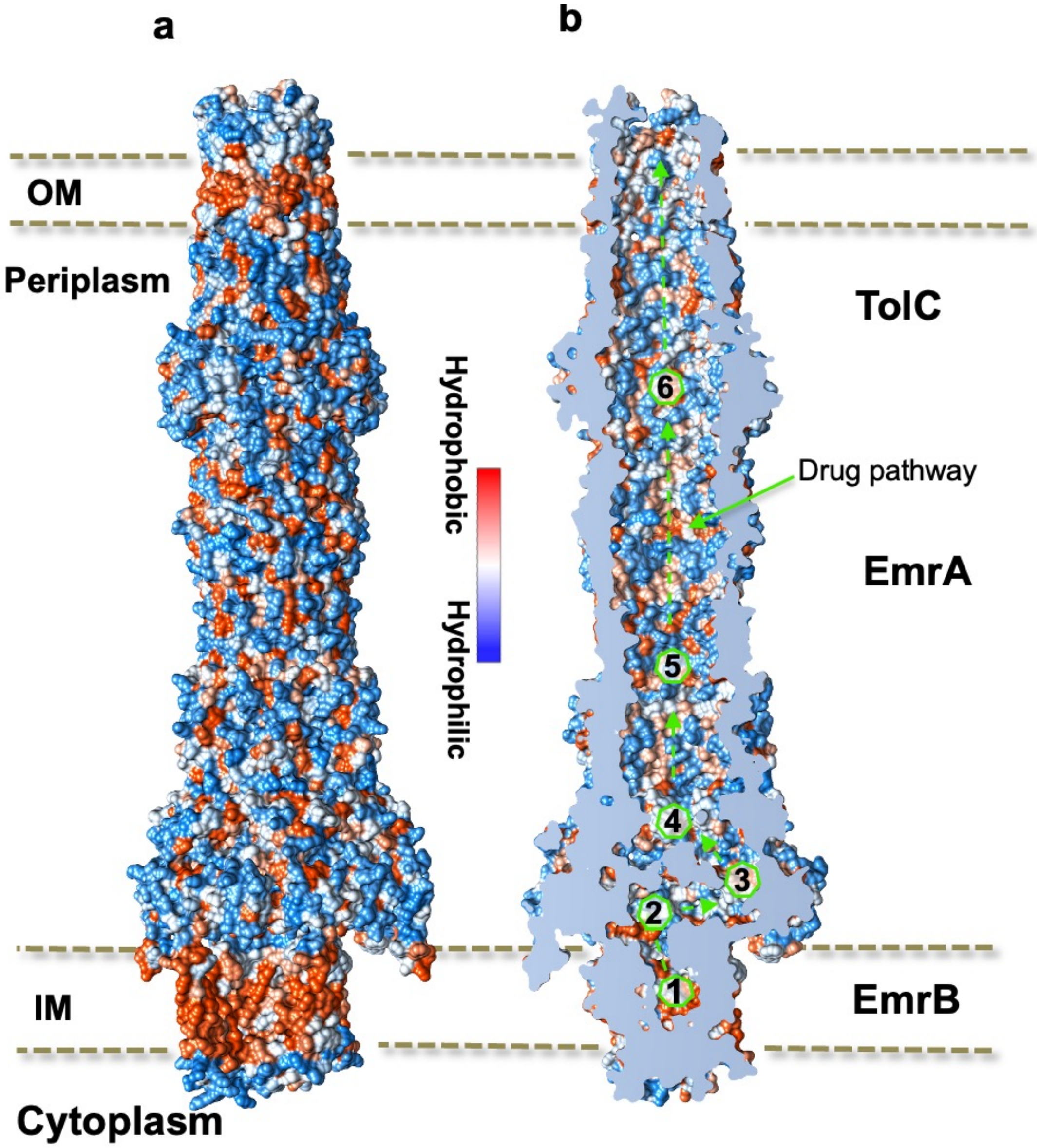

Supplementary Figure 22 | Drug transport pathway of EmrAB-TolC. a Surface representation and **b** sliced view of EmrAB-TolC. Residues are colored based on hydrophobicity. The surface of EmrB Cavity$_{out}$ is more hydrophobic than the other parts of the channel. The entire transport pathway comprises [1] EmrB Cavity$_{out}$, [2] EmrA β-barrel Cavity, [3] β-CL1PH, [4] EmrA lipoyl chamber, [5] EmrA α-helical barrel nanochannel, and [6] TolC α-helical barrel nanochannel, which form a sealed channel.

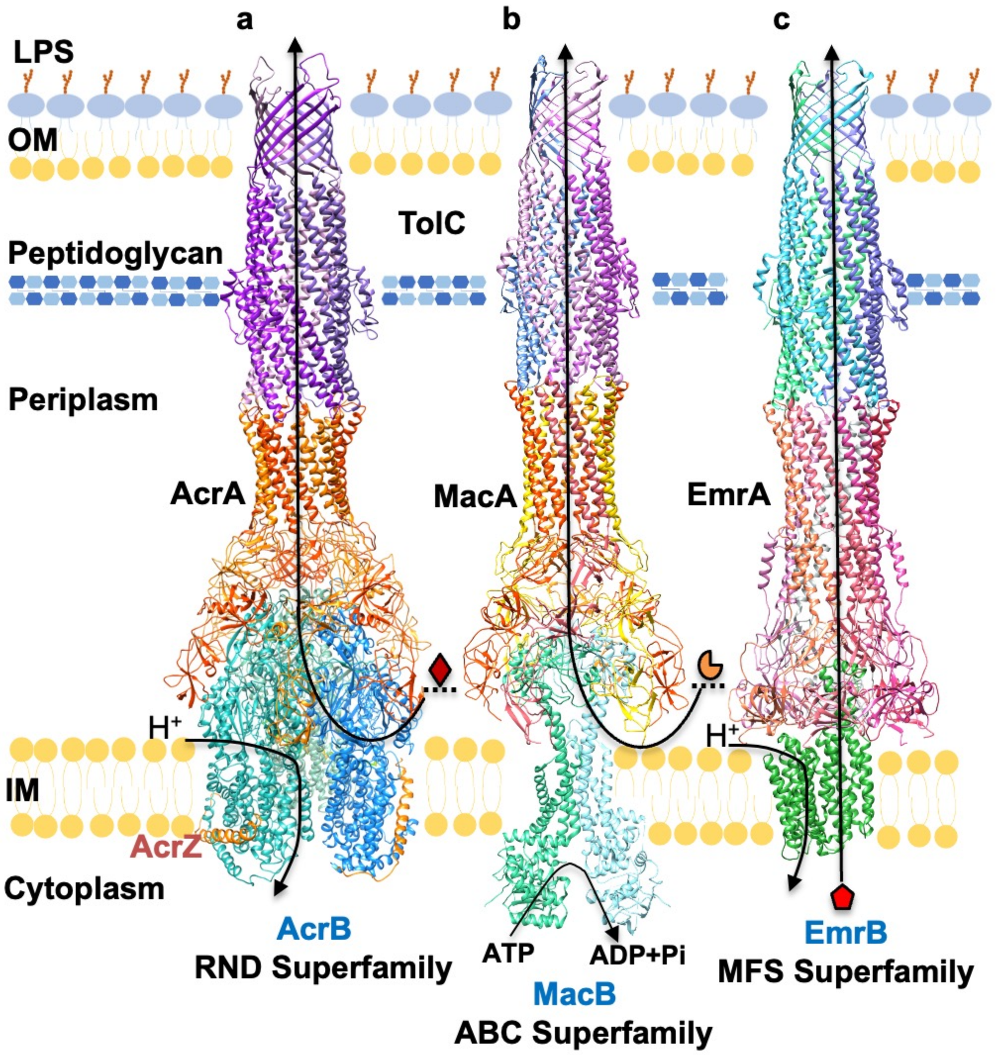

Supplementary Figure 23 | Schematic diagrams of representative structures of tripartite multidrug efflux pumps. The cell envelope of Gram-negative bacteria is made up of two layers of phospholipids: the inner membrane (IM) and the outer membrane (OM). The OM is distinct as it has an asymmetric structure, with a layer of lipopolysaccharide (LPS) in the outer leaflet. These two membranes are separated by the periplasm, a gel-like space that contains a thin layer of peptidoglycan. Three classes of tripartite efflux pumps are known to span this cell envelope: RND types exemplified by AcrAB-TolC (**a**) (PDB code: 5O66), ABC types represented by MacAB-TolC (**b**) (PDB code: 5NIK), and MFS types such as EmrAB-TolC (**c**). The RND-type AcrAB-TolC and the ABC-type MacAB-TolC pumps utilize a two-step mechanism to recognize substrates at the outer leaflet of the IM and periplasm and efflux them to the cell exterior. By contrast, the MFS-type EmrAB-TolC pump employs a one-step mechanism to directly transport substrates from the inner leaflet of the IM and cytoplasm to the cell exterior.

Supplementary Table 1.

Primers used to generate the constructs

Primer Name	Primer Sequence (5′–3′)
Petduet_F	TCCAGATCAGCCAAAGGCGTCATGCCCATGGTATATCTCCTTCTTAAAG
Petduet_R	GCCTCGAGCACCACCACCACCACCACCACCACCACTGAGCG
brilpet-F	GGCATGACGCCTTTGGCTGATCTGGAAGACAATTGGGAAACTCTGAACGAC
bril-link-R	AGGGTGCTCCTGGCCCTCTCCAGGTACTTCTGAATGTATGCATTCCGGGTGGTC
emrB-link-F	AGGGCCAGGAGCACCCTGATGGTCATTATGACGATTGCGCTGTCACTGGC
emrBGS-R	CATGGAGCCGCCACCCCCAGCAAACCACACCAGCCCCAGCAG
emrAGS-F	GGGGGTGGCGGCTCCATGAGCGCAAATGCGGAGACTCAAACC
emrApet-R	TGGTGGTGCTCGAGGCCAGCGTTAGCTTTTACGATATCGTCGATC
TolCinf_F	AAGGAGATATACATATGAAGAAATTGCTCCCCATTCTTATCGGCC
TolCFLAGXhol_R	GAGCTCGAGTCACTTATCGTCGTCATCCTTGTAATCGTTACGGAAAGGGTTATGA CCGTTACTGGT
TolCFLAG_inf_R	TTGAGATCTGCCATATGTCACTTATCGTCGTCATCCTTGTAATCGTTACG
emrAinsert_F	CTTTAATAAGGAGATATACCATGGATGAACAAAAACAGAGGGTTTAC
emrAinsert_R	AAAAGCTAACGCTGGCTAAGGATCCGAATTCGAGCTCGGCGC
*Δ*emrAinsert_F	TTTAATAAGGAGATATACCCACTTCGAAGAAACCGATGACGCATACGTG
acrAs_F	TTTAATAAGGAGATATACCATGAACAAAAACAGAGGGTTTACGCCTCTG
acrAs_R	GTTTCTTCGAAGTGTTTGTCGTCACATCCTGTTAGGGCTAAGCTGC
EmrAB_F	AAGGAGATATACCATGGGCATGAGCGCAAATGCGGAGACTCAAACCCCGC
EmrAB_R	GCATTATGCGGCCGCTCATTAGTGCGCACCGCCTCCGCCGC
Petduet_V_F	TGAGCGGCCGCATAATGCTTAAGTCGA
Petduet_V_R	GCCCATGGTATATCTCCTTCTTAAAGTTAAACAAAATTATTTCTAGAG
$emrB_{D29N}$_F	CGACATTCATGCAGGTGCTGAACTCCACCATTGCTAACGTGGCGA
$emrB_{D29N}$_R	TCGCCACGTTAGCAATGGTGGAGTTCAGCACCTGCATGAATGTCG
$emrB_{N62A}$_F	TAATCACTTCTTTCGGGGTGGCGGCAGCCATCTCGATCCCGCTTACCGGC
$emrB_{N62A}$_R	GCCGGTAAGCGGGATCGAGATGGCTGCCGCCACCCCGAAAGAAGTGATTA
$emrB_{R109A}$_F	AGCAGCCTGAATATGCTGATCTTCTTCGCGGTGATTCAGGGGATTGTCGCCG
$emrB_{R109A}$_R	CGGCGACAATCCCCTGAATCACCGCGAAGAAGATCAGCATATTCAGGCTGCT
$emrB_{V147A}$_F	GCGCTGGCGTTGTGGTCGATGACGGCGATTGTCGCGCCAATTTGCGGCCCGA
$emrB_{V147A}$_R	TCGGGCCGCAAATTGGCGCGACAATCGCCGTCATCGACCACAACGCCAGCGC
$emrB_{V148A}$_F	CTGGCGTTGTGGTCGATGACGGTGGCGGTCGCGCCAATTTGCGGCCCG
$emrB_{V148A}$_R	CGGGCCGCAAATTGGCGCGACCGCCACCGTCATCGACCACAACGCCAG
$emrB_{I288A}$_F	CGTATATGCTCTACTTCGGCGCTGCGGTTCTGCTGCCGCAGTTGTTGCA
$emrB_{I288A}$_R	TGCAACAACTGCGGCAGCAGAACCGCAGCGCCGAAGTAGAGCATATACG
$emrA_{L302Q/L303Q}$_F	CTGGATATGGGCACAGGTAGCGCGTTCTCACAGCAACCAGCGCAAAATGCGACC GGTAACTGGATC
$emrA_{L302Q/L303Q}$_R	GATCCAGTTACCGGTCGCATTTTGCGCTGGTTGCTGTGAGAACGCGCTACCTGT GCCCATATCCAG
$emrA_{I313Q/V315T/V316T}$ _F	CTTCCAGCGCAAAATGCGACCGGTAACTGGCAGAAAACGACTCAGCGTCTGCCT GTGCGTATCGAACTGGAC
$emrA_{I313Q/V315T/V316T}$ _R	GTCCAGTTCGATACGCACAGGCAGACGCTGAGTCGTTTTCTGCCAGTTACCGGT CGCATTTTGCGCTGGAAG

Supplementary Table 2.

Data collection, processing, and model building

	**EmrAB-TolC pump-EA**<br>PDB ID: 8ZAL<br>EMDB ID: EMD-39879	**EmrAB-TolC pump-FA**<br>PDB ID: 8ZAR<br>EMDB ID: EMD-39885
**Data collection and processing**		
Magnification	45,871.6	47,169.9
Voltage (kV)	300	300
Electron exposure ($e^-/Å^2$)	60	56.6
Defocus range (μm)	-1, -2.5	-1, -2.5
Pixel size (Å)	1.09	1.06
Symmetry imposed	C1	C1
Initial particle images (no.)	816,894	762,142
Final particle images (no.)	19,180	18,440
Map resolution (Å, FSC = 0.143)	3.13	3.59
Particle box size	400	400
**Refinement**		
**Model composition**		
Chains	10	10
Atoms	59223 (Hydrogens: 29683)	59993 (Hydrogens: 30115)
Protein residues	3841	3880
**Bonds (RMSD)**		
Bond lengths (Å)	0.004 (0)	0.005 (0)
Bond angles (°)	0.528 (0)	1.000 (0)
**Validation**		
MolProbity score	1.26	1.38
Clash score	2.65	3.75
Map/Model CC (Å, FSC=0.5)	3.15	3.84
**Ramachandran plot**		
Favored (%)	97.33	97.15
Allowed (%)	2.64	2.80
Outliers (%)	0.03	0.05
