# Supplementary figures and images for: A model for drug transport across two membranes of Gram-negative bacteria by an MFS tripartite assembly
Source: Nat Commun. 2026 Mar 16;17:4039. doi: 10.1038/s41467-026-70500-5 (PMC13139468; doi:10.1038/s41467-026-70500-5)

## Slide 1
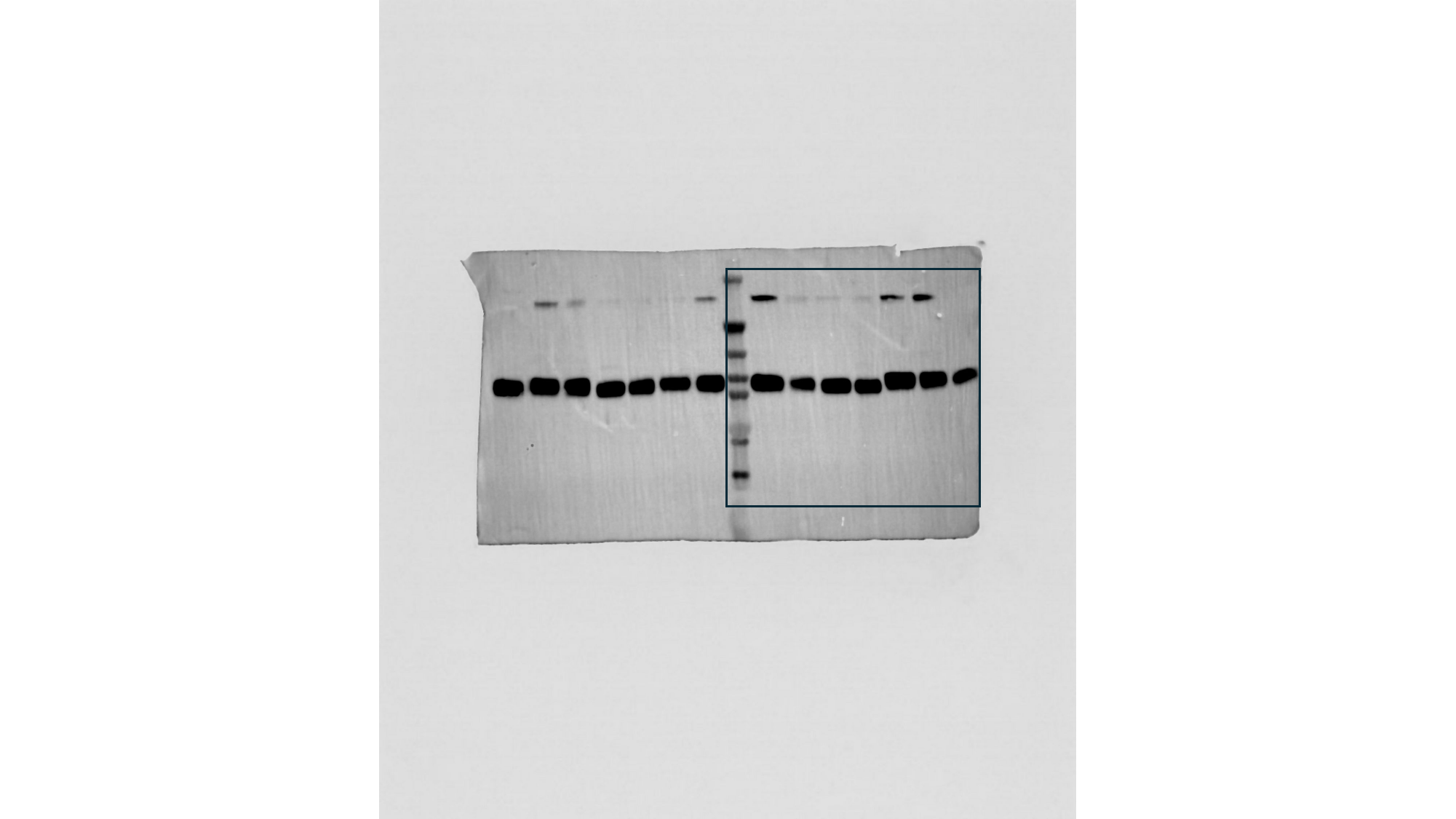

Supplement: Supplementary file 4 — Source data [file 41467_2026_70500_MOESM4_ESM.zip › crop_western_4g.pptx]

## Slide 1
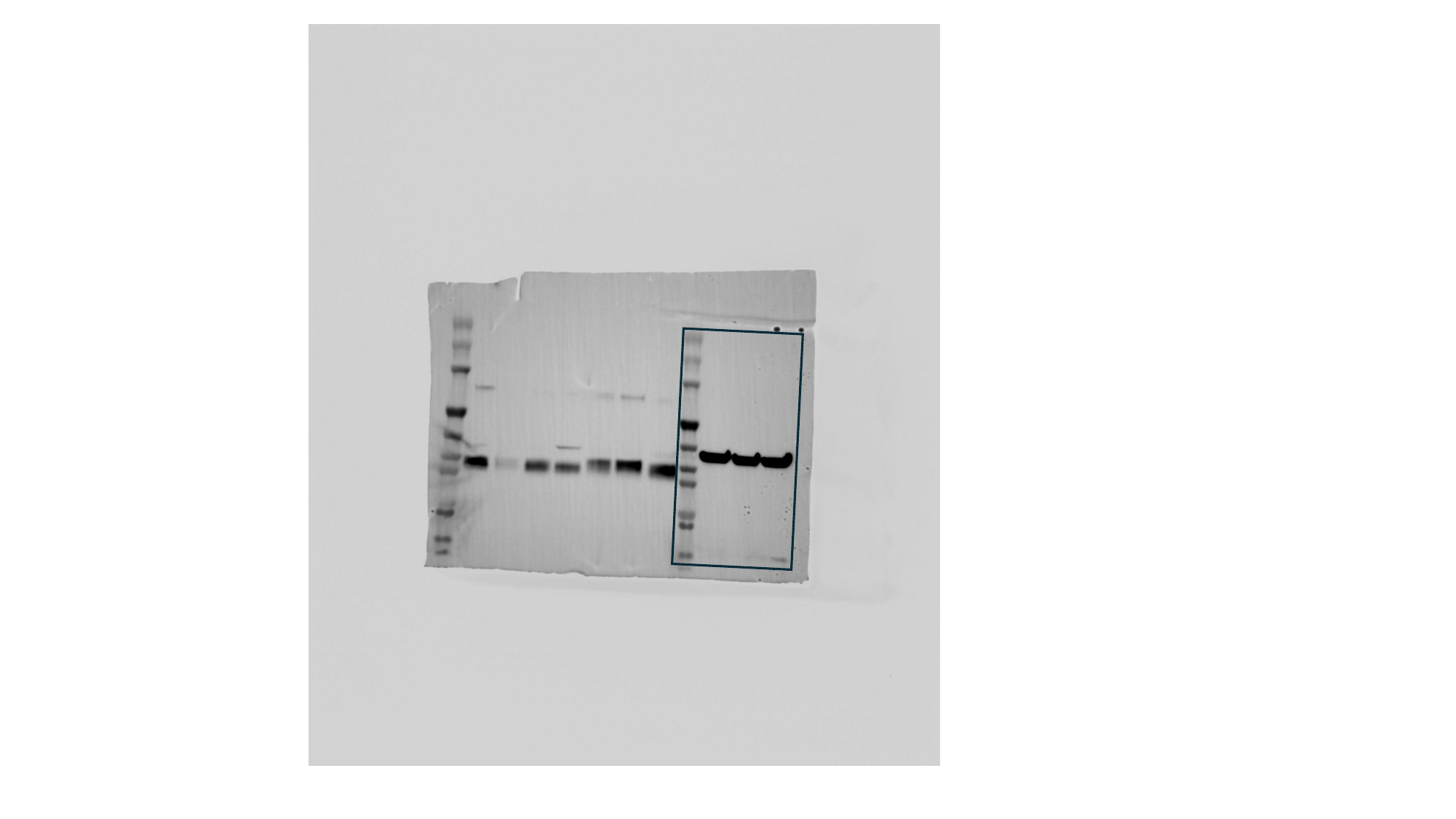

Supplement: Supplementary file 4 — Source data [file 41467_2026_70500_MOESM4_ESM.zip › crop_western_3e.pptx]
